# Changes in the healthfulness of food and beverage purchases from 2006 to 2022 by outlet type in Mexico

**DOI:** 10.1186/s12916-025-04036-8

**Published:** 2025-04-07

**Authors:** Ana Paula Domínguez-Barreto, Irene Farah, Nancy López-Olmedo, Carolina Pérez-Ferrer, Yenisei Ramírez-Toscano, Brent A. Langellier, M. Arantxa Colchero, Juan A. Rivera-Dommarco, Tonatiuh Barrientos-Gutiérrez, Dalia Stern

**Affiliations:** 1 Center for Research on Population Health, National Institute of Public Health, Cuernavaca, Morelos Mexico; 2 Center for Research on Nutrition and Health, National Institute of Public Health, Cuernavaca, Morelos, Mexico; 3 Department of Urban and Regional Planning, University of Illinois at Urbana-Champaign, Champaign, IL USA; 4 Dornsife School of Public Health, Drexel University, Philadelphia, PA USA; 5 Center for Research on Health Systems, National Institute of Public Health, Cuernavaca, Morelos Mexico; 6 SECIHTI - Center for Research on Population Health, National Institute of Public Health, Cuernavaca, Morelos Mexico

**Keywords:** Food purchases, Healthfulness, Retail food environment, ENIGH, Processing levels, Urbanicity, Education, Mexico

## Abstract

**Background:**

To better inform retail food environment policies in the global south, it is necessary to further understand the healthfulness of food and beverages purchased by type of food outlet over time.

**Methods:**

Using repeated cross-sectional data from the National Income and Expenditure Survey (ENIGH) in Mexico (2006 to 2022), we estimate the percentage of food and beverage purchases by processing level for each food outlet for the overall population and stratify by education (proxy of socioeconomic status) and urbanicity levels.

**Results:**

In 2006, the food outlets with the largest proportions of ultra-processed foods purchases were chain convenience stores (49%), small neighborhood stores (37%), and supermarkets (35%). In contrast, the outlets with the highest proportions of minimally processed food purchases were street markets (83%), public markets (81%), and specialty stores (75%). Over time, households increased the proportion of expenditure in minimally processed foods in supermarkets and slightly in small neighborhood stores (49 to 54% and 46 to 47%, respectively). Conversely, the proportion of expenditures in minimally processed foods decreased from 70 to 62% in street vendors. Households without formal education and residing in rural areas increased their minimally processed food purchases in specialty stores, but decreased in street vendors, acquaintances, and public markets. Households with higher education and residing in more urbanized areas increased their purchases of minimally processed foods in supermarkets and small neighborhood stores and decreased in street vendors. These households also increased their purchases in ultra-processed foods in chain convenience stores.

**Conclusions:**

There is a wide range of food outlets in Mexico, each with varying levels of healthfulness. While purchases in supermarkets have become healthier, particularly among higher socioeconomic households and in larger cities, small neighborhood stores have also shown improvements, especially in lower-income households and smaller cities. Since no outlet exclusively sells healthy or unhealthy foods, policies should focus on where people make the majority of their purchases and address healthfulness variations based on education level education and urbanization.

**Supplementary Information:**

The online version contains supplementary material available at 10.1186/s12916-025-04036-8.

## Background

Previous studies have established a connection between the retail food environment and health outcomes in Mexico. Specifically, they have highlighted the rise in supermarkets and chain-convenience stores, as well as the increase in the availability of ultra-processed foods within stores, as contributing factors to the prevalence of obesity and diet-related chronic diseases [1–7]. In countries of the global north, studies have shown that most food purchases happen in supermarkets and mass-merchandisers [8, 9]. Yet, the retail food environment in the global south is more heterogenous than in the global north [10, 11]. Despite the increased penetration of outlets such as supermarkets and chain convenience stores in the global south [1], a wide variety of traditional food retailers prevail. Small neighborhood stores, specialty stores, and public markets still predominate [12, 13], and the presence of the informal food sector is ubiquitous [10, 11, 13, 14]. See Table 1 for food and beverage outlet characteristics.

**Table 1:**
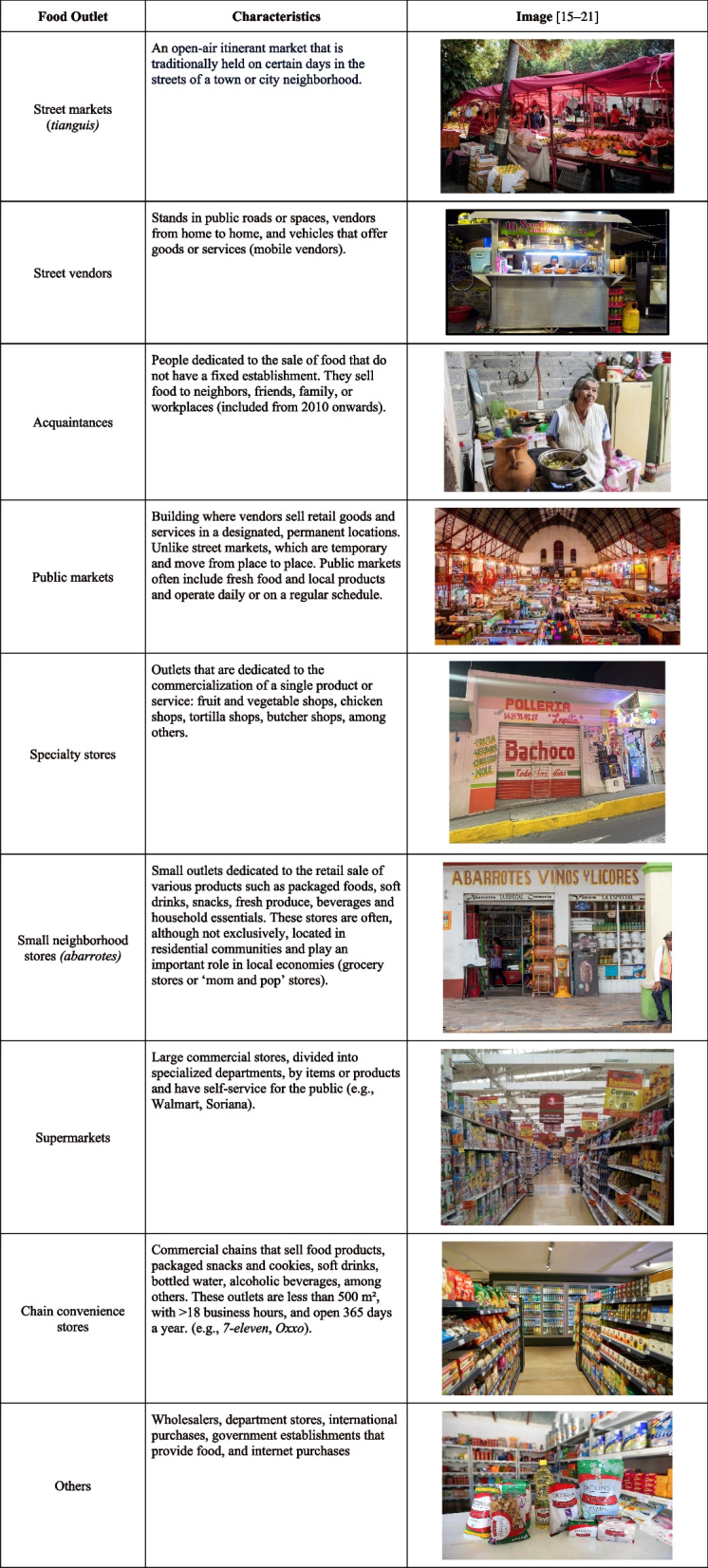
Food and beverage outlet characteristics [15–21]

Recent studies have tried to better understand the contribution of the different food outlets to total food purchases in Mexico [22, 23]. For example, in 2020, traditional stores represented 71.6% of total food purchases, followed by outlets in the informal food sector (13.7%), supermarkets (10.0%), convenience stores (1.3%) and other outlets (1.4%). This research also shows how food purchases vary by household education levels and the size of cities where people live. For instance, households in rural areas and small cities, and those with lower education levels, have made most of their food purchases in small neighborhood stores over time. These households have shown the greatest increase in food purchases from chain convenience stores and specialty stores. Street vendors account for most of the food purchases in the informal sector, especially in rural areas and among households with lower educational levels. However, these have decreased over time, similarly to food purchases in public markets [23]. In contrast, households in metropolitan areas and those with higher education levels have increasingly turned to supermarkets, specialty stores, and small neighborhood stores, maintaining an important proportion of their food purchases in supermarkets over time. Another study showed that most unhealthy foods were purchased at small neighborhood stores and supermarkets, while healthy foods were predominantly purchased at markets and specialty stores [24]. Yet, little is known about the changes in the healthfulness of food purchases in the different food outlets *over time* [1–6]. Moreover, it is unclear whether changes in the healthfulness of food purchases in different food outlets differ by education and urbanicity levels.

Several high-profile initiatives have implemented policies targeting the food environment, including the retail food environment, to improve diet quality and address global secular increases in diet-related chronic diseases (e.g., INFORMAS) [25, 26]. However, the evidence regarding these policies is largely focused on the global north [26, 27]. It is unclear whether policies designed and tested in the global north will be contextually relevant for the global south. Other international initiatives like the Hungry Cities Partnership highlight the role of informal outlets in shaping food purchases across different countries and the relevance of the informal food sector within retail food environments [28]. Yet, with some exceptions [29], most of them lack an analysis of the level of healthfulness of these outlets. Thus, to better inform retail food environment policies to ensure access to healthy foods for the entire population, accounting for social and geographic inequalities, it is imperative to understand how different types of food retailers contribute to food purchases.

To address these gaps, we used the National Income and Expenditure Survey (ENIGH) from 2006 to 2022 to describe the trends in food and beverage purchases by processing level for each outlet type for the general population, and by education and urbanicity levels. Since the global food production and distribution systems have changed food consumption with the rise of neoliberalism [30–33], we hypothesize that supermarkets, convenience stores, and small neighborhood stores have become unhealthier. In contrast, we argue that specialty stores, public markets, and informal food outlets have experienced fewer changes in healthfulness since they offer mostly minimally processed foods [24]. We also hypothesize that as households in urban areas and those with higher education levels typically have greater access to supermarkets and chain convenience stores, it may lead to a shift towards processed and ultra-processed foods due to the prevalence of these types of outlets. In contrast, households in rural areas and those with lower education levels will tend to purchase more minimally processed foods, since they rely more on traditional food outlets like specialty stores, public markets, and street vendors [22].

## Methods

### Data sources

We used nine rounds of the National Income and Expenditure Survey (*Encuesta Nacional de Ingresos y Gastos de los Hogares*, ENIGH): 2006, 2008, 2010, 2012, 2014, 2016, 2018, 2020, 2022, which is publicly available [34]. We selected these years because the distinction of outlets prior to 2006 is not as detailed, limiting our understanding of the healthfulness of food and beverage purchasing patterns in different establishments.

The survey is conducted every 2 years by the National Institute of Statistics and Geography of Mexico (INEGI), with every round of the survey being representative at the national level. Starting in 2016, the survey also became representative at the state level. ENIGH collects information on household income and expenditure, sociodemographic characteristics, and characteristics of the household members, among others [35]. Households’ information on daily expenses in ENIGH was collected for seven consecutive days. Household expenditure in foods and beverages was reported by the household member responsible for the purchases. Additionally, each member of the household kept a food diary. The food diary included the name of the food/beverage, quantity purchased (liters or kilograms), the price paid (MXN), and the food outlet where purchases happened [35].

In rounds 2006 to 2022, ENIGH included a total of 430,545 households with a variability of 20,330 households in 2006 to 90,102 in 2022. We excluded households that did not report purchases on food or beverages, or households that only reported purchases in restaurants, cafes, bars, and low-budget restaurants (*n* = 5461, 1.27%), given that we do not know what was purchased. The number of households that reported food purchases by food outlets can be found in Additional File 1: Table 1. The final analytical sample included 425,084 households.

### Food and beverage outlets

Over the years, ENIGH has categorized Mexico’s food and beverage outlets differently. In 2006 and 2008, ENIGH classified outlets in 15 categories: public markets; street markets (*tianguis*); street vendors; small neighborhood stores (*abarrotes*); specialty stores; low-budget restaurants; restaurants, cafes, bars; chain convenience stores; supermarkets; department stores; membership stores; purchases out of the country; and others. Starting in 2010 and up to 2022, ENIGH added four new outlets: acquaintances; internet purchases; and two government food establishments, Diconsa (subsidized basic food and household products) and Liconsa (subsidized milk and milk). These were added to the existing categories to better reflect the diversity of points of sale in Mexico.

Considering that food outlet categorization varies over time, we classified outlets into 9 mutually exclusive categories (Table 1): (1) street markets (*tianguis*); (2) street vendors; (3) acquaintances; (4) public markets; (5) specialty stores; (6) small neighborhood stores (*abarrotes*); (7) supermarkets; (8) chain convenience stores; and (9) others. Others include food outlet categories that individually accounted for a small percentage of the total food and beverage expenditure (< 1%). Public markets, specialty stores, and small neighborhood stores have been central to Mexico´s food environment and are considered “traditional” food outlets. Street markets, street vendors, and acquaintances are also considered traditional outlets, however they are informal establishments. As the monitoring of informal outlets is more complex compared to other outlets, their description in food retail literature is still limited. Finally, supermarkets and chain convenience stores are characterized as food outlets that have risen in the past few decades and belong to the formal sector.

### Processing level of food and beverage purchases

We used the processing level of foods and beverages according to the NOVA classification criteria [36] as a reference measure of the healthfulness of food purchases [37]. Since 2006, food and beverages in ENIGH have been classified into 247 items, of which 234 items were classified according to their processing level (Additional File 2: Table 2). The excluded items were purchases of food for animals, tobacco-related products, expenses related to food preparation, and food provided by the government. We classified the 234 items as (1) unprocessed and minimally processed foods, including foods that have not been transformed in any way, such as fresh fruits, vegetables, legumes, grains, and unprocessed meat; (2) processed culinary ingredients, including ingredients used to add flavor to food, such as oils, fats, salt, sugar, and spices; (3) processed foods, including foods that go through a transformation process like canned, cured, and smoked; and (4) ultra-processed foods, which are products manufactured from highly complex industrial procedures and have added sweeteners, flavors, and other additives. Some examples include sugary drinks, packaged snacks, and candy.

### Education and urbanicity

We used education and urbanicity levels to get a better understanding of the diversity of food environments across geographies and socioeconomic strata, since place is crucial to understand people’s health [38] and their relationship with food [39].

We used the highest education level of the head of the household as a proxy for the household’s socioeconomic status. Since education is correlated to income [40] and we wanted to use a consistent measurement over time [41, 42], we used education instead of income because income is often underreported [43, 44], fails to accurately capture the richest and poorest households [45], and is subject to a bias that varies over time, with current estimates underestimating income more than in the past [44]. ENIGH considers eleven categories of education level: (1) without formal education, (2) preschool, (3) incomplete primary school, (4) complete primary school, (5) incomplete middle school, (6) complete middle school, (7) incomplete high school, (8) complete high school, (9) incomplete professional degree, (10) complete professional degree, (11) postgraduate degree. For this analysis, we grouped them into 4 mutually exclusive categories: (1) without formal education (1); (2) preschool or primary school (2 to 5); (3) middle school or high school (6 to 9); and (4) higher education (10 to 11).

Urbanicity is defined by INEGI according to the number of inhabitants: (1) rural areas are those with less than 2500 inhabitants; (2) small cities include localities with a population between 2500 and 14,999 inhabitants; (3) medium-sized cities are those with a population between 15,000 to 99,999 inhabitants; and (4) large cities (metropolitan cities) include localities with more than 100,000 inhabitants [35].

### Statistical analysis

For every survey year, we estimated the percentage of food and beverage expenditure (hereafter purchases) by processing level for each outlet for the overall population and stratifying by education and urbanicity. Descriptive statistics were prepared. As a hypothetical example, results should be interpreted as follows: for a given household, out of every $100 MXN spent in street vendors, 80% was spent on minimally processed foods, and 20% of ultra-processed foods. Households that did not report food or beverage expenses in a specific outlet were excluded from the analysis. All analyses were conducted in Stata 16 using the SVY command to account for the complex survey design and weighed to generate nationally representative estimates. Weights were created for every ENIGH survey to account for the selection probabilities and survey non-response to match the estimated population for every survey year from the [35].

## Results

Table 2 shows the sociodemographic characteristics of households which reported food and beverage purchases in the ENIGH surveys from 2006 to 2022. The proportion of households without formal education and primary school decreased over time, while households with high school and higher education levels increased from 2006 to 2022. The proportion of households living in rural areas slightly increased from 2020 to 2022.

**Table 2:** Households’ sociodemographic characteristics: the National Income and Expenditure Survey (ENIGH), 2006–2022

Year	2006	2008	2010	2012	2014	2016	2018	2020	2022
**Total households (*N*)**	20,330	29,042	27,224	8854	19,266	69,476	73,838	87,970	89,102
**Education level, %**^**a**^									
Without formal education	9.9	9.4	9.0	9.0	7.7	7.1	6.7	6.3	5.7
Preschool/primary school	57.0	44.7	42.6	40.0	38.1	36.3	34.5	33.6	32.0
Middle/high school	22.1	35.1	36.7	39.8	41.5	43.6	45.0	46.0	47.0
Higher education	10.9	10.8	11.8	11.3	12.7	13.0	13.8	14.0	15.3
**Urbanicity, %**									
Rural areas	22.3	21.4	21.4	22.0	22.0	21.7	23.1	21.5	23.0
Small cities	13.2	13.8	13.8	13.3	13.5	13.9	14.1	13.7	13.9
Medium-sized cities	14.7	14.5	14.4	14.4	14.8	14.5	14.7	14.9	14.6
Metropolitan cities	49.8	50.3	50.4	50.3	49.8	49.9	48.1	49.9	48.5

^a^Education level of the head of the household

### Trends in food and beverage purchases by food outlet

**Fig. 1:**
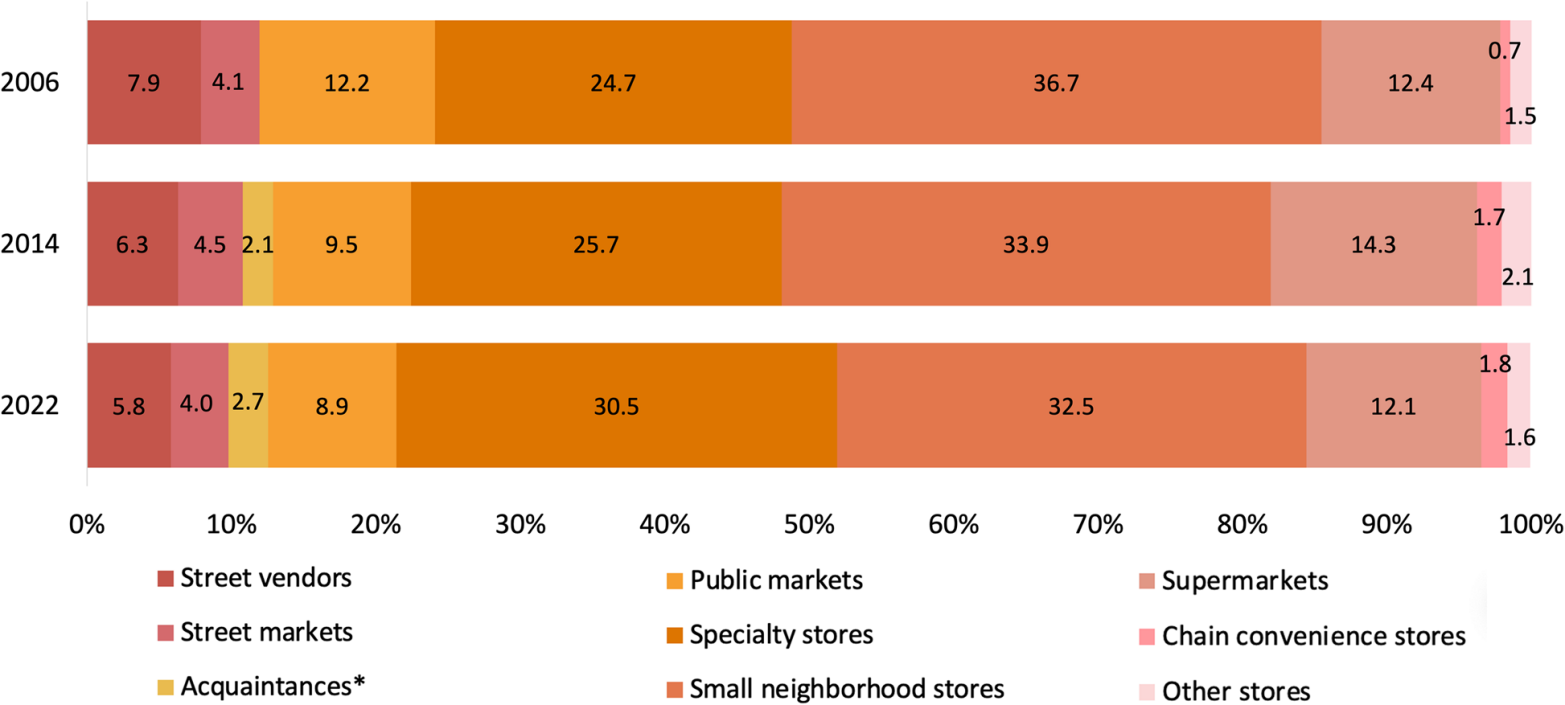
Proportion of food and beverage purchases (% of the total purchases) by outlet type in 2006, 2014, and 2022, ENIGH. *Acquaintances are included in ENIGH from 2010 onwards. Other outlets include wholesalers, department stores, international purchases, government establishments that provide food, and internet purchases

Figure 1 shows the proportion of food purchases (% of the total expenditure) by food outlet type in 2006, 2014, and 2022. Overall, we found a clear trend in each of the food outlets, except in supermarkets and street markets. For supermarkets, the proportion of food purchases peaked in 2014 at 14.3% and decreased back to the baseline percentage (12.1%). For street markets, the proportion of food purchases increased to 4.5% in 2014 and then decreased to 4.0%, slightly lower than in 2006. In 2006, most food purchases among Mexican households were carried out in small neighborhood stores with 36.7%, followed by specialty stores with 24.7% and 12.2% in public markets. In 2022, public markets and small-neighborhood stores decreased by 3.3 percentage points (p.p.) and 4.2 p.p., respectively, while the proportion of purchases from specialty stores increased (+ 5.8 p.p.). Moreover, food purchases in chain convenience stores showed an increase from 2006 to 2022 (+ 1.1 p.p.). Regarding the changes in food purchases in informal outlets (i.e., street vendors, street markets, acquaintances), in 2006, these accounted for 11.9% of the total food purchases, by 2022, there was a decrease of 2.1 p.p. in street vendors, while purchases at street markets remained stable over time. Information on acquaintances was included in ENIGH starting 2010, when food purchases in these outlets accounted for 2.1% of the total purchases and showed a slight increase of 0.6 p.p. by 2022.

### Trends in food and beverage purchases by processing level and by food outlet

**Fig. 2:**
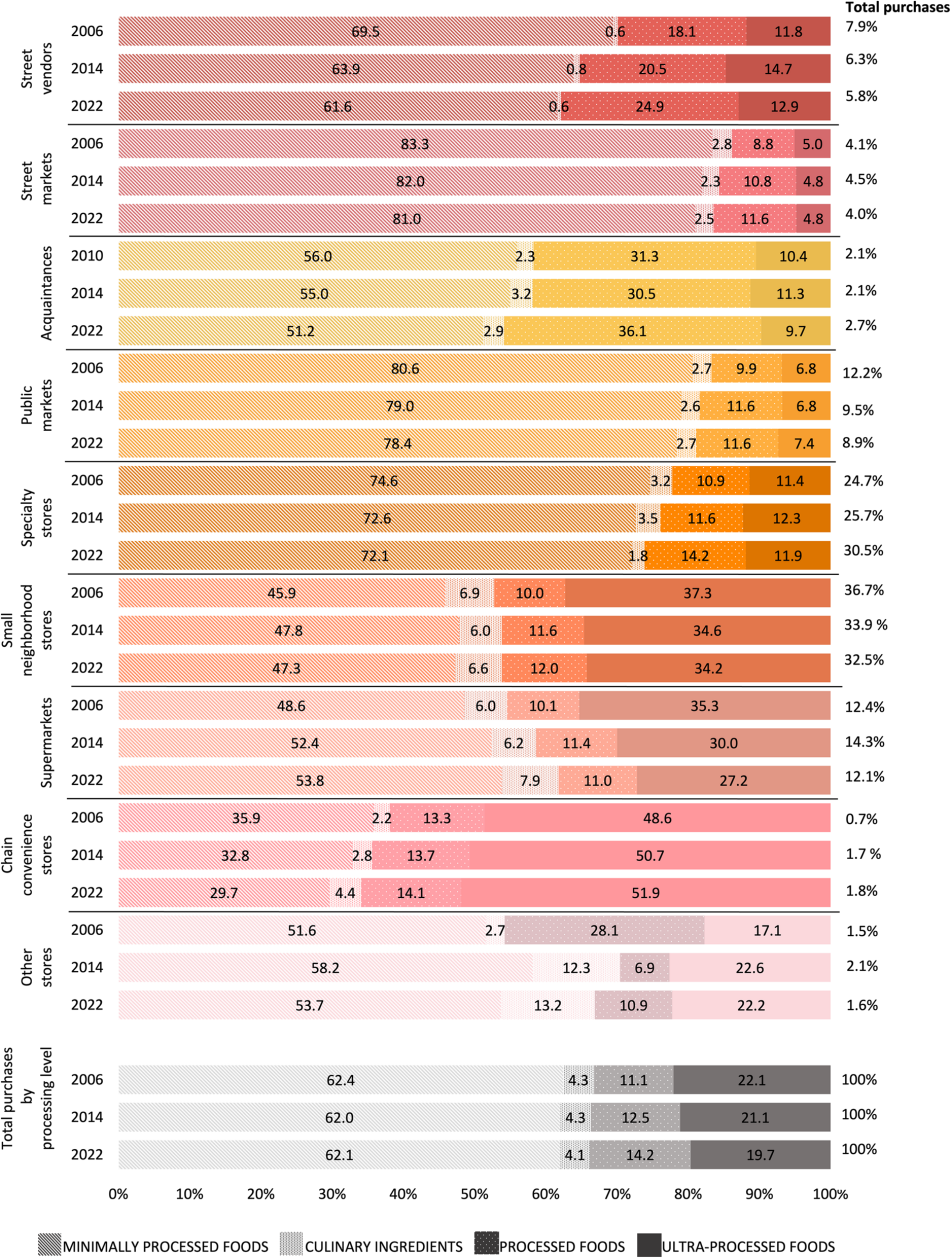
Proportion of food and beverage purchases by processing level and outlet type in 2006, 2014, and 2022, ENIGH. The values on the right show the distribution of household food and beverage purchases by type of food outlet in the corresponding years. Other outlets include wholesalers, department stores, international purchases, government establishments that provide food, and internet purchases. Acquaintances are included in ENIGH from 2010 onwards

Figure 2 shows the proportion of purchases (% of expenditure) of foods and beverages of different processing levels by food outlet type for selected years (2006, 2014, 2022). Yearly purchases can be found in Additional file 3: Table 3. In 2006, the outlets with the highest proportion of minimally processed foods purchases were street markets (83.3%), public markets (80.6%), specialty stores (74.6%), and street vendors (69.5%). In contrast, the highest percentage of ultra-processed foods was purchased at chain convenience stores (48.6%), small neighborhood stores (37.3%)*,* and supermarkets (35.3%).

By 2014, minimally processed food purchases declined in most outlets (except for small neighborhood stores, supermarkets, and other stores), with the largest decreases observed in street vendors (− 5.6 p.p.), convenience stores (− 3.1 p.p.), and specialty stores (− 2.0 p.p.). Compared to 2006, by 2022, purchases of minimally processed foods decreased by 4.8 p.p. in acquaintances, 6.2 p.p. in chain convenience stores, and 7.9 p.p. in street vendors. Street markets, public markets, and specialty stores only presented a slight decrease in the purchases of these products. These decreases were accompanied by an increase in processed food purchases in specialty stores (+ 3.3 p.p.), acquaintances (+ 4.8 p.p.), and street vendors (+ 6.8 p.p.). The shift toward processed foods was more pronounced in 2014, before stabilizing somewhat in 2022. Between 2006 and 2014, ultra-processed food purchases rose in street vendors, (+ 2.9 p.p.), acquaintances (+ 0.9 p.p.), specialty stores (+ 0.9 p.p.), and other stores (+ 5.5 p.p.) before declining again in 2022. In contrast, ultra-processed food purchases decreased by 3.1 p.p. in small neighborhood stores*,* and 8.1 p.p. in supermarkets; and purchases of minimally processed foods increased by 5.2 p.p. in supermarkets. The proportion of ultra-processed food purchases remained stable at chain convenience stores.

### Trends in food and beverage purchases by processing level and by food outlet, stratified by education

Table 3 shows the proportion in households’ food purchases by processing level and by food outlets, stratified by education level of the head of the household, in 2006, 2014, and 2020. Percentage data from all ENIGH rounds (2006 to 2022) are available in Additional file 4: Tables 4.1, 4.2, and 4.3. The proportion of food purchases by processing level across the different education levels were similar to the ones observed in the overall sample; however, households without formal education had higher purchases of minimally processed foods in all food outlets, compared to households with higher education. From 2006 to 2022, households without formal education increased their purchases of minimally processed foods in specialty stores (+ 3.6 p.p.). The purchases of these foods also increased in supermarkets (+ 6.1 p.p.) and small neighborhood stores (+ 3.5 p.p.) in 2014, but decreased again by 2022, showing similar estimates to those in 2006. These households decreased their minimally processed foods purchases in chain convenience stores (− 20.5 p.p.), street vendors (− 5.4 p.p.), public markets (− 2.5 p.p.), and in acquaintances from 2014 to 2022 (− 3.5 p.p.). Households without formal education also decreased their purchases of ultra-processed foods in chain convenience stores (− 13.3 p.p.) and supermarkets (− 3.8 p.p.). The decline in supermarkets was more pronounced in 2014, after which the trend appears to be stabilizing again. A similar pattern was observed in small neighborhood stores. Additionally, these households increased their purchases of processed foods in chain convenience stores (+ 19.7 p.p.), and street vendors (+ 5.9 p.p.).

**Table 3:** Trends in food and beverage purchases by processing level and by food outlet, stratified by education (ENIGH 2006, 2014, 2022). Data for acquaintances is not available for ENIGH of 2006

Food outlet	Food processing level	Education level											
		Without formal education			Preschool/primary school			Middle school/high school			Higher education		
		2006	2014	2022	2006	2014	2022	2006	2014	2022	2006	2014	2022
		Mean percentage (SD)											
Street vendors	Minimally processed	**75.4** (1.8)	**68.5** (1.9)	**70.0** (1.2)	**70.0** (1.1)	**68.0** (1.1)	**66.4** (0.6)	**63.7** (1.6)	**61.0** (1.4)	**58.6** (0.6)	**71.4** (1.8)	**55.4** (2.3)	**55.0** (1.1)
	Culinary ingredients	**0.9** (0.3)	**1.8** (0.6)	**0.7** (0.1)	**0.6** (0.2)	**0.9** (0.2)	**0.6** (0.1)	**0.5** (0.2)	**0.5** (0.1)	**0.5** (0.1)	**0.3** (0.2)	**1.1** (0.7)	**0.5** (0.1)
	Processed	**11.9** (1.4)	**16.5** (1.6)	**17.8** (1.0)	**17.2** (0.7)	**17.0** (0.8)	**20.9** (0.5)	**23.5** (1.3)	**22.9** (1.0)	**27.3** (0.5)	**19.5** (1.5)	**28.5** (2.1)	**31.4** (1.0)
	Ultra-processed	**11.8** (1.4)	**13.2** (1.3)	**11.5** (0.8)	**12.1** (1.7)	**14.2** (1.7)	**12.1** (0.4)	**12.2** (0.9)	**15.6** (0.8)	**13.7** (0.4)	**8.7** (1.1)	**15.0** (1.6)	**13.2** (0.7)
Street markets	Minimally processed	**85.9** (1.6)	**85.6** (1.9)	**84.1** (1.2)	**82.4** (0.9)	**82.5** (0.9)	**81.0** (0.6)	**84.3** (1.5)	**81.3** (1.1)	**80.4** (0.6)	**84.7** (2.2)	**81.1** (2.2)	**82.1** (1.1)
	Culinary ingredients	**3.0** (0.6)	**3.3** (1.1)	**3.6** (0.5)	**3.3** (0.3)	**2.8** (0.4)	**3.3** (0.3)	**2.2** (0.5)	**2.1** (0.3)	**2.2** (0.2)	**1.0** (0.3)	**0.9** (0.2)	**1.6** (0.2)
	Processed	**7.1** (1.2)	**7.8** (1.4)	**8.0** (0.9)	**9.0** (0.6)	**9.8** (0.7)	**11.2** (0.6)	**8.6** (1.0)	**11.6** (0.9)	**12.3** (0.6)	**9.9** (1.8)	**13.4** (2.0)	**11.8** (0.9)
	Ultra-processed	**4.0** (0.7)	**3.3** (0.9)	**4.2** (0.6)	**5.3** (0.6)	**4.9** (0.5)	**4.5** (0.3)	**5.0** (0.8)	**5.0** (0.6)	**5.1** (0.3)	**4.4** (1.5)	**4.6** (0.9)	**4.5** (0.6)
Acquaintances	Minimally processed	**-**	**63.8** (3.2)	**60.3** (1.6)	**-**	**57.9** (1.7)	**55.2** (0.8)	**-**	**49.5** (1.9)	**48.2** (0.8)	**-**	**49.4** (4.1)	**43.1** (1.7)
	Culinary ingredients	**-**	**7.2** (1.7)	**6.5** (0.8)	**-**	**3.9** (0.8)	**3.9** (0.3)	**-**	**1.4** (0.4)	**1.9** (0.2)	**-**	**1.7** (0.9)	**1.1** (0.3)
	Processed	**-**	**19.4** (2.3)	**25.3** (1.4)	**-**	**27.9** (1.4)	**32.2** (0.7)	**-**	**35.8** (1.8)	**39.3** (0.7)	**-**	**38.9** (3.7)	**45.2** (1.7)
	Ultra-processed	**-**	**9.7** (2.0)	**8.0** (0.8)	**-**	**10.2** (0.9)	**8.7** (0.4)	**-**	**13.3** (1.3)	**10.7** (0.5)	**-**	**10.1** (2.5)	**10.6** (0.9)
Public markets	Minimally processed	**82.1** (1.4)	**81.8** (1.7)	**79.6** (1.1)	**80.6** (0.7)	**79.2** (0.8)	**78.0** (0.5)	**79.1** (0.9)	**78.3** (0.8)	**77.9** (0.4)	**81.8** (1.2)	**79.4** (1.4)	**80.4** (0.7)
	Culinary ingredients	**4.2** (0.8)	**2.8** (0.5)	**3.1** (0.4)	**2.8** (0.3)	**3.3** (0.3)	**3.3** (0.2)	**2.1** (0.2)	**2.0** (0.2)	**2.5** (0.1)	**1.4** (0.2)	**2.1** (0.4)	**1.8** (0.2)
	Processed	**7.2** (0.9)	**9.1** (1.2)	**10.8** (1.0)	**9.5** (0.4)	**10.8** (0.6)	**11.5** (0.4)	**12.2** (0.8)	**12.6** (0.6)	**11.7** (0.3)	**9.9** (0.9)	**12.5** (1.1)	**11.4** (0.6)
	Ultra-processed	**6.5** (1.0)	**6.2** (0.9)	**6.5** (0.6)	**7.0** (0.4)	**6.7** (0.5)	**7.2** (0.3)	**6.6** (0.5)	**7.2** (0.4)	**7.9** (0.2)	**6.9** (0.8)	**6.1** (0.7)	**6.5** (0.4)
Specialty stores	Minimally processed	**74.9** (1.9)	**73.4** (1.8)	**78.5** (0.7)	**76.6** (0.6)	**75.7** (0.7)	**76.2** (0.3)	**73.9** (0.9)	**72.4** (0.6)	**71.6** (0.3)	**63.6** (1.3)	**63.8** (1.2)	**63.0** (0.5)
	Culinary ingredients	**11.5** (1.7)	**11.2** (1.6)	**4.8** (0.4)	**3.3** (0.3)	**5.3** (0.6)	**2.9** (0.2)	**0.6** (0.1)	**1.4** (0.2)	**1.1** (0.1)	**0.4** (0.1)	**0.4** (0.1)	**0.6** (0.1)
	Processed	**7.0** (0.8)	**7.4** (0.7)	**9.1** (0.4)	**10.0** (0.4)	**9.6** (0.4)	**11.8** (0.2)	**12.5** (0.6)	**12.6** (0.4)	**14.6** (0.2)	**16.1** (1.0)	**16.9** (0.9)	**19.5** (0.4)
	Ultra-processed	**6.5** (0.6)	**8.0** (0.9)	**7.6** (0.4)	**10.1** (0.4)	**9.5** (0.4)	**9.1** (0.2)	**13.0** (0.6)	**13.5** (0.4)	**12.6 (**0.2)	**19.9** (1.1)	**19.0** (1.0)	**17.0** (0.4)
Small neighborhood stores	Minimally processed	**50.0** (0.9)	**53.5** (1.0)	**50.8** (0.5)	**46.9** (0.5)	**50.0** (0.5)	**49.4** (0.3)	**42.9** (0.7)	**45.8** (0.5)	**46.0** (0.2)	**41.8** (1.0)	**43.3** (1.1)	**44.8** (0.5)
	Culinary ingredients	**11.0** (0.6)	**9.1** (0.5)	**8.9** (0.3)	**7.4** (0.2)	**6.9** (0.2)	**7.8** (0.1)	**4.9** (0.4)	**5.0** (0.2)	**5.9** (0.1)	**3.4** (0.3)	**4.1** (0.5)	**4.9** (0.2)
	Processed	**8.1** (0.5)	**10.1** (0.6)	**10.1** (0.3)	**9.6** (0.2)	**11.0** (0.3)	**11.2** (0.1)	**11.4** (0.4)	**12.3** (0.3)	**12.3** (0.1)	**10.7** (0.6)	**12.1** (0.7)	**13.4** (0.3)
	Ultra-processed	**30.9** (0.9)	**27.4** (1.0)	**30.1** (0.5)	**36.0** (0.5)	**32.2** (0.5)	**31.6** (0.3)	**40.8** (0.6)	**36.8** (0.4)	**35.9** (0.2)	**44.1** (1.0)	**40.4** (1.1)	**37.0** (0.5)
Supermarkets	Minimally processed	**51.2** (3.4)	**57.3** (3.1)	**51.7** (2.0)	**48.6 (**1.0)	**53.4** (1.0)	**53.5** (0.6)	**46.6** (1.1)	**51.6** (0.7)	**53.3** (0.4)	**50.6** (1.0)	**52.5** (1.1)	**55.4** (0.6)
	Culinary ingredients	**13.9** (2.5)	**11.3** (2.0)	**13.1** (1.3)	**7.0** (0.5)	**7.4** (0.4)	**10.0** (0.4)	**5.0** (0.3)	**6.5** (0.3)	**8.1** (0.2)	**4.2** (0.3)	**3.7** (0.3)	**5.3** (0.2)
	Processed	**6.0** (1.2)	**9.3** (1.6)	**10.2** (1.4)	**9.5** (0.5)	**9.8** (0.5)	**10.5** (0.4)	**10.7** (0.6)	**11.3** (0.4)	**10.9** (0.2)	**11.2** (0.6)	**13.6** (0.7)	**11.9** (0.3)
	Ultra-processed	**28.8** (2.8)	**22.1** (2.6)	**25.0** (1.7)	**34.9** (0.9)	**29.4** (0.9)	**25.9** (0.5)	**37.7** (1.1)	**30.6** (0.7)	**27.7** (0.4)	**34.0** (0.9)	**30.2** (0.9)	**27.5** (0.5)
Chain convenience stores	Minimally processed	**52.8** (18.2)	**41.3** (10.3)	**32.3** (3.7)	**37.8** (3.6)	**35.8** (2.5)	**32.6** (1.2)	**37.0** (3.3)	**31.0** (1.5)	**28.8** (0.7)	**32.7** (3.1)	**33.0** (2.1)	**29.1** (1.0)
	Culinary ingredients	**1.1** (1.1)	**6.5** (3.4)	**15.1** (3.5)	**2.9** (2.0)	**4.8** (1.3)	**5.8** (0.6)	**2.6** (1.0)	**2.6** (0.5)	**4.5** (0.4)	**1.2** (0.5)	**1.3** (0.3)	**2.7** (0.3)
	Processed	**0.2** (0.2)	**9.4** (4.3)	**19.9** (4.2)	**15.0** (2.8)	**13.2** (2.3)	**14.4** (1.0)	**11.4** (1.8)	**14.3** (1.1)	**14.2** (0.6)	**14.2** (2.1)	**13.6** (1.5)	**13.4** (0.8)
	Ultra-processed	**46.0** (18.4)	**42.8** (9.6)	**32.7** (4.2)	**44.2** (3.5)	**46.2** (2.6)	**47.1** (1.3)	**49.0** (3.1)	**52.2** (1.7)	**52.5** (0.8)	**51.9** (3.3)	**52.1** (2.0)	**54.8** (1.0)
Other stores	Minimally processed	**57.0** (3.6)	**58.6** (3.4)	**58.3** (2.0)	**56.0** (2.1)	**59.3** (1.8)	**56.5** (1.2)	**40.9** (3.0)	**59.1** (2.5)	**54.0** (1.2)	**41.2** (3.7)	**49.9** (3.9)	**44.4** (1.8)
	Culinary ingredients	**5.1** (1.5)	**17.8** (2.5)	**23.2** (1.6)	**2.9** (0.5)	**13.3** (1.2)	**17.2** (0.9)	**1.2** (0.4)	**11.3** (1.3)	**10.6** (0.6)	**1.7** (0.5)	**4.6** (1.4)	**4.9** (0.7)
	Processed	**26.8** (4.1)	**2.7** (0.8)	**5.1** (0.8)	**26.9** (1.9)	**5.8** (0.9)	**7.1** (0.6)	**34.6** (2.8)	**8.5** (1.1)	**10.9** (0.8)	**24.3** (3.1)	**11.2** (2.2)	**22.2** (1.7)
	Ultra-processed	**11.0** (3.3)	**20.9** (2.9)	**13.3** (1.4)	**14.3** (1.2)	**21.6** (1.4)	**19.1** (0.9)	**23.4** (2.4)	**21.1** (1.8)	**24.5** (1.0)	**32.9** (3.2)	**34.3** (3.3)	**28.5** (1.7)

*SD* standard deviation

Other stores include wholesalers, department stores, international purchases, government establishments that provide food, and internet purchases

Households with higher education increased their purchases of minimally processed foods in supermarkets (+ 4.8 p.p.) and small neighborhood stores (+ 3.0 p.p.) and decreased their purchases of minimally processed foods in street vendors (− 16.4 p.p.). The purchases of the same type of foods decreased in acquaintances (− 6.3 p.p.) from 2014 to 2022. Households with higher education also decreased their purchases of ultra-processed foods in supermarkets (− 6.5 p.p.), small neighborhood stores (− 7.1 p.p.), and specialty stores (− 2.9 p.p.), and increased their purchases of processed (+ 11.9 p.p.) and ultra-processed foods (+ 4.5 p.p.) in street vendors from 2006 to 2022. Ultra-processed foods peaked in 2014 with 15.0% of the food purchases from households with higher education in these outlets. In chain convenience stores, these households increased their ultra-processed foods purchases by 2.9 p.p. and decreased in minimally processed foods by 3.6 p.p.

### Trends in food and beverage purchases by processing level and by food outlet, stratified by urbanicity

Table 4 shows the proportion in households’ food purchases by processing level and by food outlets, stratified by urbanicity, in 2006, 2014, and 2020. Percentage data from all ENIGH rounds (2006 to 2022) are available in Additional file 5: Tables 5.1, 5.2, and 5.3. The proportion of food purchases by processing level in rural areas and cities were similar to the results described for the overall sample. Households residing in rural areas increased their purchases of minimally processed foods in supermarkets (+ 4.6 p.p.), chain convenience stores (+ 5.0 p.p.), and specialty stores (+ 3.3 p.p.), and decreased their purchases of minimally processed foods in street vendors (− 3.8 p.p.), acquaintances (− 3.7 p.p. from 2010 to 2022) and public markets (− 3.0 p.p.). Households residing in rural areas decreased their purchases of ultra-processed foods in supermarkets (− 8.4 p.p.) chain convenience stores (− 16.9 p.p.), and increased purchases of processed foods in chain convenience stores (+ 5.3 p.p.) and public markets (+ 2.0 p.p.). In street vendors, ultra-processed food purchases increased 3.4 p.p. in rural areas and 2.8 p.p. in metropolitan cities from 2006 to 2014 and decreased again in 2022 (− 2.9 p.p. and − 1.6 p.p. respectively).

**Table 4:** Trends in food and beverage purchases by processing level and by food outlet, stratified by urbanicity (ENIGH 2006, 2014, 2022). Data for acquaintances is not available for ENIGH of 2006

Food outlet	Food processing level	Urbanicity											
		Rural areas			Small cities			Medium-sized cities			Metropolitan cities		
		2006	2014	2022	2006	2014	2022	2006	2014	2022	2006	2014	2022
		Mean percentage (SD)											
Street vendors	Minimally processed	**76.1** (1.6)	**71.7** (1.5)	**72.3** (0.6)	**70.5** (3.7)	**67.4** (2.1)	**64.6** (1.1)	**69.6** (1.5)	**61.0** (2.3)	**56.9** (1.3)	**64.5** (1.0)	**58.5** (1.4)	**54.3** (0.7)
	Culinary ingredients	**0.9** (0.2)	**1.4** (0.3)	**0.9** (0.1)	**1.2** (0.6)	**0.5** (0.2)	**0.5** (0.1)	**0.4** (0.1)	**0.7** (0.3)	**0.4** (0.1)	**0.3** (0.1)	**0.6 (0.2)**	**0.3** (0.1)
	Processed	**11.6** (1.2)	**12.1** (1.0)	**14.9** (0.4)	**14.2** (1.9)	**17.7** (1.4)	**21.7** (0.9)	**18.9** (1.2)	**22.0** (1.6)	**27.5** (1.0)	**23.7** (0.8)	**26.6** (1.1)	**32.6** (0.7)
	Ultra-processed	**11.4** (0.9)	**14.8** (1.0)	**11.9** (0.4)	**14.2** (2.5)	**14.3** (1.4)	**13.1** (0.7)	**11.1** (0.9)	**16.3** (1.4)	**15.3** (0.9)	**11.5** (0.6)	**14.3** (0.9)	**12.7** (0.5)
Street markets	Minimally processed	**83.4** (1.8)	**81.9** (1.7)	**80.5** (0.7)	**86.2** (3.2)	**82.2** (1.8)	**81.5** (0.9)	**82.4** (1.6)	**83.8** (1.8)	**82.4** (1.2)	**83.0** (0.8)	**81.6** (0.9)	**80.8** (0.6)
	Culinary ingredients	**5.0** (0.8)	**4.5** (0.9)	**4.7** (0.3)	**3.0** (0.1)	**2.9** (0.8)	**4.1** (0.5)	**2.6** (0.5)	**2.5** (0.7)	**2.1** (0.3)	**2.1** (0.3)	**1.6** (0.2)	**1.7** (0.1)
	Processed	**5.4** (0.6)	**7.4** (1.0)	**9.0** (0.4)	**6.9** (1.4)	**10.6** (1.6)	**10.2** (1.0)	**10.6** (1.4)	**8.0** (1.0)	**10.8** (1.2)	**9.8** (0.6)	**12.3** (0.8)	**12.9** (0.5)
	Ultra-processed	**6.2** (1.3)	**6.2** (1.0)	**5.9** (0.4)	**3.9** (1.4)	**4.3** (0.8)	**4.2** (0.5)	**4.3** (0.6)	**5.7** (1.3)	**4.7** (0.6)	**5.0** (0.5)	**4.5** (0.5)	**4.7** (0.3)
Acquaintances	Minimally processed	-	**63.3** (2.0)	**62.4** (0.8)	-	**61.9** (2.5)	**54.9** (1.3)	-	**60.1** (3.7)	**48.1** (1.7)	-	**36.2** (2.2)	**35.5** (1.1)
	Culinary ingredients	-	**6.3** (1.2)	**5.2** (0.4)	-	**2.1** (1.1)	**2.6** (0.5)	-	**0.8** (0.3)	**1.3** (0.3)	-	**1.1** (0.3)	**0.9** (0.2)
	Processed	-	**21.4** (1.6)	**24.1** (0.7)	-	**25.9** (2.2)	**32.8** (1.3)	-	**25.9** (3.0)	**42.6** (1.6)	-	**48.7** (2.1)	**51.2** (1.1)
	Ultra-processed	-	**9.0** (1.1)	**8.3** (0.4)	-	**10.2** (1.5)	**9.7** (0.6)	-	**13.1** (2.0)	**8.1** (0.7)	-	**14.0** (1.5)	**12.4** (0.7)
Public markets	Minimally processed	**82.2** (1.8)	**78.3** (1.3)	**79.2** (0.5)	**81.7** (1.7)	**78.7** (1.8)	**78.0** (0.9)	**80.0** (1.0)	**81.7** (1.2)	**78.9** (0.6)	**79.9** (0.6)	**78.4** (0.7)	**78.1** (0.4)
	Culinary ingredients	**4.6** (0.7)	**4.1** (0.5)	**3.9** (0.2)	**3.1** (0.6)	**3.0** (0.5)	**2.1** (0.2)	**2.4** (0.3)	**2.1** (0.3)	**2.2** (0.2)	**2.0** (0.1)	**2.1** (0.2)	**2.6** (0.1)
	Processed	**7.3** (1.0)	**9.0** (0.9)	**9.1** (0.4)	**7.1** (0.8)	**11.5** (1.5)	**12.9** (0.7)	**11.0** (0.7)	**10.7** (0.9)	**12.7** (0.5)	**11.2** (0.4)	**12.7** (0.6)	**11.6** (0.3)
	Ultra-processed	**5.9** (1.0)	**8.6** (0.9)	**7.9** (0.4)	**8.1** (1.5)	**6.8** (0.9)	**7.1** (0.5)	**6.6** (0.6)	**5.4** (0.7)	**6.2** (0.4)	**7.0** (0.3)	**6.7** (0.4)	**7.7** (0.2)
Specialty stores	Minimally processed	**72.9** (1.9)	**70.3** (1.7)	**76.2** (0.4)	**79.4** (1.6)	**77.9** (1.2)	**77.5** (0.5)	**77.8** (1.0)	**75.9** (1.2)	**73.8** (0.5)	**72.8** (0.5)	**70.9** (0.6)	**68.3** (0.3)
	Culinary ingredients	**10.0** (1.4)	**13.0** (1.6)	**5.6** (0.4)	**5.9** (1.3)	**4.1** (0.9)	**1.7** (0.2)	**0.8** (0.1)	**1.0** (0.2)	**0.8** (0.1)	**0.4** (0.0)	**0.6** (0.1)	**0.6** (0.0)
	Processed	**8.4** (1.0)	**7.8** (0.6)	**10.1** (0.2)	**7.9** (0.6)	**9.4** (0.7)	**11.6** (0.3)	**10.8** (0.7)	**11.2** (0.7)	**14.4** (0.4)	**12.8** (0.4)	**13.8** (0.4)	**16.6** (0.2)
	Ultra-processed	**8.7** (1.0)	**8.8** (0.7)	**8.1** (0.2)	**6.8** (0.8)	**8.6** (0.7)	**9.2** (0.3)	**10.6** (0.6)	**11.9** (0.9)	**11.0** (0.3)	**14.0** (0.4)	**14.8** (0.5)	**14.6** (0.2)
Small neighborhood stores	Minimally processed	**48.8** (0.8)	**49.1** (0.8)	**49.4** (0.3)	**49.2** (1.5)	**50.2** (0.8)	**49.7** (0.5)	**45.8** (0.8)	**50.5** (1.0)	**48.3** (0.5)	**43.5** (0.4)	**45.6** (0.5)	**45.0** (0.3)
	Culinary ingredients	**11.5** (0.6)	**9.2** (0.4)	**9.0** (0.1)	**9.0** (0.6)	**7.1** (0.4)	**7.4** (0.2)	**6.5** (0.6)	**5.0** (0.3)	**6.3** (0.2)	**4.1** (0.1)	**4.4** (0.2)	**5.0** (0.1)
	Processed	**8.4** (0.4)	**9.7** (0.4)	**10.7** (0.2)	**9.2** (0.8)	**12.4** (0.6)	**12.1** (0.2)	**9.8** (0.4)	**11.5** (0.6)	**11.6** (0.2)	**11.0** (0.2)	**12.3** (0.3)	**12.7** (0.2)
	Ultra-processed	**31.3** (0.8)	**31.9** (0.8)	**30.9** (0.3)	**32.5** (1.2)	**30.4** (0.8)	**30.8** (0.4)	**37.8** (0.6)	**32.9** (0.9)	**33.8** (0.5)	**41.4** (0.4)	**37.7** (0.5)	**37.2** (0.3)
Supermarkets	Minimally processed	**43.7** (3.5)	**47.7** (1.8)	**48.3** (0.7)	**31.9** (3.6)	**50.8** (2.0)	**48.7** (1.3)	**47.6** (1.4)	**51.6** (1.2)	**53.9** (0.9)	**50.4** (0.6)	**53.4** (0.6)	**55.3** (0.4)
	Culinary ingredients	**11.3** (2.1)	**11.6** (0.9)	**12.8** (0.5)	**9.9** (2.6)	**7.6** (1.1)	**11.8** (1.0)	**6.3** (0.5)	**6.9** (0.5)	**8.3** (0.6)	**5.1** (0.2)	**5.2** (0.2)	**6.7** (0.2)
	Processed	**6.9** (0.9)	**9.3** (0.7)	**9.3** (0.4)	**14.9** (3.3)	**10.3** (0.9)	**11.2** (0.7)	**9.6** (0.7)	**11.6** (1.1)	**10.7** (0.4)	**10.3** (0.3)	**11.7** (0.4)	**11.3** (0.2)
	Ultra-processed	**38.0** (4.2)	**31.4** (1.7)	**29.6** (0.6)	**43.3** (2.5)	**31.2** (1.8)	**28.3** (1.0)	**36.5** (1.2)	**29.9** (1.2)	**27.1** (0.7)	**34.2** (0.6)	**29.7** (0.6)	**26.7** (0.3)
Chain convenience stores	Minimally processed	**27.2** (7.5)	**32.8** (4.1)	**32.2** (1.4)	**45.8** (18.0)	**37.3** (5.5)	**31.4** (2.1)	**38.1** (5.4)	**33.1** (3.4)	**30.9** (1.4)	**35.6** (2.2)	**32.6** (1.3)	**29.0** (0.6)
	Culinary ingredients	**1.3** (0.7)	**1.7** (1.0)	**7.9** (1.0)	**0.0** (0.0)	**6.1** (4.1)	**6.8** (1.1)	**7.0** (4.9)	**2.8** (1.0)	**4.5** (0.6)	**1.6** (0.5)	**2.7** (0.5)	**3.8** (0.3)
	Processed	**9.2** (4.8)	**11.0** (2.9)	**14.5** (1.1)	**31.7** (16.2)	**9.7** (2.4)	**14.2** (1.5)	**7.8** (2.6)	**16.2** (3.7)	**14.5** (1.6)	**13.8** (1.4)	**13.8** (0.9)	**13.9** (0.5)
	Ultra-processed	**62.3** (9.3)	**54.6** (4.6)	**45.4** (1.6)	**22.5** (12.8)	**46.9** (6.2)	**47.6** (2.1)	**47.1** (4.9)	**47.9** (4.3)	**50.0** (1.8)	**49.1** (2.2)	**51.0** (1.3)	**53.3** (0.7)
Other stores	Minimally processed	**64.3** (1.9)	**53.6** (1.8)	**52.8** (0.9)	**64.9** (6.1)	**66.6** (3.8)	**62.7** (2.4)	**45.9** (4.3)	**74.0** (4.9)	**54.8** (2.8)	**37.2** (2.0)	**58.4** (2.8)	**51.6** (1.5)
	Culinary ingredients	**3.7** (0.8)	**17.7** (1.4)	**22.3** (0.8)	**3.4** (1.1)	**8.9** (2.8)	**7.8** (1.0)	**1.3** (0.5)	**5.2** (1.9)	**5.9** (1.5)	**2.0** (0.4)	**4.8** (0.9)	**3.5** (0.5)
	Processed	**21.1** (2.6)	**6.1** (0.8)	**5.8** (0.3)	**20.1** (4.0)	**4.1** (1.1)	**10.5** (1.6)	**32.2** (4.4)	**3.2** (1.1)	**13.6** (2.1)	**35.8** (2.0)	**11.4** (1.8)	**17.8** (1.1)
	Ultra-processed	**10.9** (2.3)	**22.6** (1.5)	**19.1** (0.6)	**11.6** (2.6)	**20.4** (2.8)	**19.1** (1.7)	**20.6** (3.0)	**17.6** (3.8)	**25.8** (2.6)	**25.0** (1.7)	**25.5** (2.3)	**27.1** (1.4)

*SD* standard deviation

Other stores include wholesalers, department stores, international purchases, government establishments that provide food, and internet purchases

Households residing in metropolitan cities increased their purchases of minimally processed foods in supermarkets (+ 4.9 p.p.) and decreased their purchases of minimally processed foods in street vendors (− 10.2 p.p.), chain convenience stores (− 6.6 p.p.) acquaintances (− 5.6 p.p. from 2010 to 2022), and specialty stores (− 4.5 p.p.). These same households decreased their purchases of ultra-processed foods in supermarkets (− 7.5 p.p.) and small neighborhood stores (− 4.2 p.p.) and increased their purchases of ultra-processed foods only in chain convenience stores by 4.2 p.p.

## Discussion

Using repeated cross-sectional data on household food purchases, we offer a detailed analysis of the healthfulness of foods and beverages purchased over time across different food outlets, including the traditional and informal sector, for the Mexican population, as well as across different education and urbanicity strata. Over time, the proportion of purchases of minimally processed foods was higher in street markets, street vendors, public markets, and specialty stores. In contrast, the highest percentage of ultra-processed foods were purchased at chain convenience stores, small neighborhood stores, and supermarkets. However, against some of our initial hypotheses, food purchases became healthier in supermarkets and less healthy in street vendors, chain convenience stores, and acquaintances. Yet, when considering the contribution of each outlet to total food purchases in Mexico, small neighborhood stores have consistently been the outlets where most of the ultra-processed foods were purchased over time, and specialty stores where most of the minimally processed foods were purchased, which appear to be increasing in households without formal education and in rural areas.

Recent studies from other countries of the global south like Vietnam or Tanzania, have found that purchases from informal vendors are primarily composed of fruits and vegetables, with relatively low purchases of ultra-processed foods [46, 47]. However, research also indicates that street vendors offer a mix of both minimally processed and ultra-processed foods [48]. Studies limited to urban areas in Mexico have also documented that street vendors have the tendency to offer ultra-processed foods [49, 50], showing that street food stands are a source of both unhealthy and healthy foods for communities across neighborhoods in Mexico City [51].

Much of the gray literature suggests that Mexico’s informal sector contributes significantly to rising rates of obesity [52, 53], often assuming that all the food that is being purchased contains unhealthy levels of saturated fats, sugar, and salt [54]. However, contrary to this assumption and as we initially hypothesized [24], our study shows that food purchases from the informal food sector, particularly from street markets, have historically been predominantly healthier. Moreover, we found that the informal sector has also provided healthy food options across all education and urban strata, being most prevalent within the most disadvantaged groups, as we initially suggested. These findings challenge the widespread belief that the informal food sector in the global south is inherently unhealthy. However, while it has historically provided healthier food options in Mexico—particularly among households without formal education and in rural areas—the observed shift toward less healthful purchasing patterns, particularly among more educated and urban households, highlights the evolving nature of food environments [2, 5, 6, 23]. Acknowledging the cultural significance, diversity, and resilience of the informal sector will be key to developing more effective, targeted public health strategies [55].

Trends in the healthfulness of purchases from traditional food outlets, such as small neighborhood stores, specialty stores, and public markets, were heterogeneous. Since we were expecting small neighborhood stores to become unhealthier [30–33], it was to our surprise that purchases in small neighborhood stores became healthier over time among households with higher education and those living in metropolitan areas. As we initially expected, household food purchases in public markets and specialty stores have been mostly represented by minimally processed foods over time. However, unexpectedly, households in metropolitan areas had a noticeable decrease in their minimally processed food purchases in specialty stores, whereas the opposite was observed in rural areas. This finding is worrisome because specialty stores are not only one of the outlets where healthier purchases are made, but the purchases in these outlets have also increased over time [23]. Moreover, even though purchases in public markets have remained healthy over time, the proportion of total food purchases from public markets has decreased over time, particularly among households with less education and those residing in rural areas [23]. Despite finding that the proportion of purchases of ultra-processed foods in small neighborhood stores has decreased over time, these outlets have accounted for the greatest proportion of food purchases in Mexico (~ 30%) [22, 23], making small neighborhood stores the outlets where people purchase the majority of ultra-processed foods.

Consistent with our findings [28] a recent study found that specialty stores and public markets were key sources of fresh food and an important food supply for poorer households, based on 2018 data. Similarly, recent studies from Mexico have found a positive association between higher densities of specialty stores with higher purchases of fruits and vegetables, lower purchases of ultra-processed foods, and better health outcomes [1, 56]. Illustrating the diversity of food outlets across geographies, these same studies found that higher densities of small neighborhood stores were associated with lower purchases of fruits and vegetables and worse health outcomes [1, 56]. These findings underscore the complexity of food environments in Mexico, highlighting the varied impacts of different food outlets on household diets. While public markets and specialty stores continue to provide healthier options, the declining role of public markets in rural areas and the shift in specialty store purchases in metropolitan areas raise concerns about access to nutritious foods. Additionally, small neighborhood stores, despite becoming somewhat healthier, still account for a disproportionate share of ultra-processed food purchases. These trends highlight the need for targeted interventions that address disparities in purchases of healthier food options, particularly among rural and less educated populations [57, 58].

Chain convenience stores and supermarkets have been an important source for unhealthy food purchases in Mexican households. While we expected food purchases from chain convenience stores to have remained unhealthy, we did not expect that purchases from supermarkets shifted towards healthier foods [30–33] (from 45.9 to 47.3% of minimally processed foods). This improvement happened across all education and urbanicity levels, but it was greater among households with higher education and those living in metropolitan cities, which represent the households with the most purchases in these outlets (19.5% and 14.5%, respectively) [22, 23]. Yet, it is relevant to emphasize that even though the number of supermarkets and chain convenience stores has increased over the years [58–61], these outlets have represented around 12% and 2% of the total food purchases over the last decade, respectively [22, 23]. This points to the need for more comprehensive public health strategies that focus not only on supermarkets and chain-convenience stores like in countries of the global north [10, 11, 55], but also on the traditional and informal food sector, which continues to dominate food purchases.

Food policy in Mexico, which includes taxes on sugar-sweetened beverages and non-essential energy dense food, as well as the implementation of front-of-pack warning labeling, has primarily focused on preventing the consumption of unhealthy products [62, 63]. While these regulations aim to shift purchasing patterns, their impact may vary across education levels, influencing how different socioeconomic groups internalize and respond to them. For instance, higher-educated households might respond more quickly to warning labels due to greater nutrition literacy [64], while price interventions like taxes might have more immediate effects in households with lower education levels [65–67]. However, our analysis did not explore these temporal relationships, which presents an important area of study for future research.

However, except for the new Mexican Dietary Guidelines [68], policies that directly promote the consumption of healthy foods at a national scale are still lacking. Retail food environments are crucial settings for promoting good nutrition, but conducting research in these contexts presents challenges due to the varied motivations of stakeholders and the complexity of retail systems. Applying some of the approaches outlined in the best practice guide recently proposed by Scapin et al. could help inform strategies for different outlets [69]. For the Mexican context, public markets and street markets represent a key opportunity to promote healthier food purchases, yet few studies have been published on how to strengthen these venues. Thus, working with local governments to create healthier retail food environments, as suggested by Scapin et al. (2025), could provide a framework for improving public markets and street markets.” For instance, improving the infrastructure and conditions of workers in public markets and street markets would benefit all socioeconomic strata since all socioeconomic levels purchase from these outlets. It would be particularly fruitful to improve markets’ maintenance, hygiene, making them more spacious, and implementing an adequate infrastructure for food conservation; characteristics that are usually lacking in these spaces [41]. Other policies could focus on spatial planning, limiting the location of chain retail outlets to promote the competition of public markets, street markets, or other smaller establishments, as has been done in Mexico City in 2011 [70]. The policies described above are challenging because they intervene directly with the private sector, limiting where and what they can sell. There is likely to be pushback from powerful trade organizations.

In terms of small neighborhood stores, policy makers could provide financial incentives or subsidies to the production of healthy foods, reducing their cost, making healthier options more accessible to both store owners and consumers, particularly in neighborhoods where households have insufficient access to healthy foods. Policy makers could also create incentive programs where small neighborhood stores can receive discounts on healthy foods if they meet specific health standards (e.g., offering a certain percentage of fresh produce or low-sugar products). Other opportunities could involve creating direct supply chains between small stores and local producers, which could increase healthy food offerings, reduce costs, and improve product freshness [71–73], as has been done through the GAIN project in Kenya [74]. Reshaping the retail food environment to promote healthy diets will require experimentation with a range of policy options that act across the food system, from producers to processors, distributors, retailers, and consumers [69, 75].

Our study is not without limitations. However, we described the healthfulness of food purchases by outlet type over time, which provides information regarding the changes in the food purchasing patterns in each outlet at a national, urbanicity, and educational level. A limitation regarding the timeframe of our study is that we were not able to see changes in the healthfulness of food purchases prior to 2006. Therefore, we cannot see the changes in the quality of food purchases in the different outlets during the late 90 s, when the modernization of the retail food environment began. Additionally, our analysis did not capture potential retailer-level changes, such as mergers, store closures, or shifts in product availability, which could influence purchasing patterns over time. Our study also did not consider the role that food prices and the diversity of outlets play in food purchasing choices, which should be included in future studies. In particular, our study period overlapped with significant economic shocks and the COVID-19 pandemic, but assessing their specific impact on purchasing patterns across different outlet types and educational groups was beyond the scope of this paper. We excluded from the analysis foods that were consumed away from home or wasted. Purchases of food consumed away from home are defined as breakfast, lunch, and dinner meals in ENIGH, representing on average, 16% of purchases from street vendors and 11% from acquaintances and are an important source of urban food provisioning. Given the difficulties of quantifying these foods, more research is needed focusing on the nutrition quality of the informal food sector [14]. It is also important to acknowledge that purchases do not equate to consumption. We were not able to account for food waste from households’ food and beverage volume records. However, the nutritional profile of purchases is highly correlated with diet quality as measured by 24-h recalls and therefore a good representation of overall intake [76], particularly when ENIGH enumerators carefully supervised the responses every day from the 7-day data collection [35]. Yet, self-reporting and recall bias may also affect the accuracy of purchase reporting, particularly for unplanned or small-scale purchases. Lastly, purchases in some outlets might be underestimated since some purchases are unplanned and households may not report them.

## Conclusions

Research on the retail food environment from the global north is not translatable to the global south. Despite the penetration of supermarkets and chain convenience stores, these are not the primary sources of food for Mexican households. To promote healthier food environments, policymakers must focus on where the majority of food purchases are made (i.e., small neighborhood, specialty stores), and where the healthiest purchases occur (i.e., street markets, public markets, and specialty stores). Additionally, interventions should target outlets where the highest proportion of ultra-processed foods are purchased (i.e., small neighborhood stores, supermarkets, and chain convenience stores). Furthermore, it is important that policymakers consider the historical context of the food environment in Mexico, including trends in spending across different types of retailers, the healthfulness of purchases at each food outlet, and understand the reasons why purchases from certain food outlets are becoming healthier, while others are becoming less healthy.

## Supplementary Information

Additional file 1. Table 1. Number of households that purchased in each food outlet from 2006 to 2022

Additional file 2. Table 2. Classification of foods from ENIGH from 2006 to 2022 according to their processing level

Additional file 3. Table 3. Trends in the proportion of food and beverage purchases by processing level by type of food outlets

Additional file 4. Table 4.1 Trends in the proportion of food and beverage purchases by processing level in informal outlets stratified by education level of the head of the household, Data from ENIGH 2006 to 2022. Table 4.2. Trends in the proportion of food and beverage purchases by processing level in traditional outlets stratified by education level of the head of the household, Data from ENIGH 2006 to 2022. Table 4.3. Trends in the proportion of food and beverage purchases by processing level in supermarkets, chain convenience stores and other outlets, stratified by education level of the head of the household, Data from ENIGH 2006 to 2022

Additional file 5. Table 5.1 Trends in the proportion of food and beverage purchases by processing level in informal outlets stratified by urbanicity, Data from ENIGH 2006 to 2022. Table 5.2. Trends in the proportion of food and beverage purchases by processing level in traditional outlets stratified by urbanicity, Data from ENIGH 2006 to 2022. Table 5.3. Trends in the proportion of food and beverage purchases by processing level in supermarkets, chain convenience stores and other outlets, stratified by urbanicity, Data from ENIGH 2006 to 2022

## Data Availability

The datasets generated and/or analyzed during the current study are available in the INEGI-ENIGH repository: https://www.inegi.org.mx/programas/enigh/nc/2022/. Datasets for each year can be found in the files below: ENIGH 2006 - Microdata: https://www.inegi.org.mx/programas/enigh/tradicional/2006/#microdatos. ENIGH 2008 - Microdata: https://www.inegi.org.mx/programas/enigh/nc/2008/#microdatos. ENIGH 2010 - Microdata: https://www.inegi.org.mx/programas/enigh/nc/2010/#microdatos. ENIGH 2012 - Microdata: https://www.inegi.org.mx/programas/enigh/nc/2012/#microdatos. ENIGH 2014 - Microdata: https://www.inegi.org.mx/programas/enigh/nc/2014/#microdatos. ENIGH 2016 - Microdata: https://www.inegi.org.mx/programas/enigh/nc/2016/#microdatos. ENIGH 2018 - Microdata: https://www.inegi.org.mx/programas/enigh/nc/2018/#microdatos. ENIGH 2020 - Microdata: https://www.inegi.org.mx/programas/enigh/nc/2020/#microdatos. ENIGH 2022 - Microdata: https://www.inegi.org.mx/programas/enigh/nc/2022/#microdatos.

## Abbreviations

INFORMAS: International Network for Food and Obesity/non-communicable Diseases Research, Monitoring and Action Support
ENIGH: National Income and Expenditure Survey (Encuesta Nacional de Ingresos y Gastos de los Hogares)
INEGI: National Institute of Statistics and Geography of Mexico (Instituto Nacional de Estadística y Geografía)
