# Supplementary material for: Changes in the healthfulness of food and beverage purchases from 2006 to 2022 by outlet type in Mexico
Source: BMC Med. 2025 Apr 7;23:205. doi: 10.1186/s12916-025-04036-8 (PMC11974062; doi:10.1186/s12916-025-04036-8)
Supplement: Supplementary file 1 — Additional file 1. Table 1. Number of households that purchased in each food outlet from 2006 to 2022 [file 12916_2025_4036_MOESM1_ESM.docx]

**Table 1. Number of households that purchased in each food outlet from 2006 to 2022**

| Food outlet | Year | | | | | | | | | |
| --- | --- | --- | --- | --- | --- | --- | --- | --- | --- | --- |
|  | 2006 | 2008 | 2010 | 2012 | 2014 | 2016 | 2018 | 2020 | 2022 | Total |
|  | Number of households | | | | | | | | | |
| Street vendors | 9,972 | 14,960 | 11,646 | 3,886 | 8,517 | 27,283 | 28,359 | 35,715 | 33,217 | 173,555 |
| Street markets | 3,040 | 4,859 | 3,971 | 1,297 | 2,890 | 8,991 | 9,796 | 10,537 | 11,278 | 56,659 |
| Acquaintances | 20,330 | 29,024 | 4,803 | 1,831 | 3,687 | 13,992 | 14,640 | 19,570 | 18,399 | 126,276 |
| Public markets | 6,269 | 9,940 | 10,273 | 2,379 | 5,434 | 16,306 | 16,747 | 17,951 | 19,320 | 104,619 |
| Specialty stores | 15,697 | 23,364 | 21,931 | 6,676 | 15,246 | 52,210 | 55,271 | 69,259 | 68,698 | 328,352 |
| Small neighborhood stores | 18,427 | 26,531 | 24,520 | 7,827 | 17,109 | 61,774 | 65,896 | 78,474 | 78,482 | 379,040 |
| Supermarkets | 5,821 | 9,384 | 8,327 | 2,616 | 6,692 | 23,427 | 23,484 | 25,526 | 28,273 | 133,550 |
| Chain-convenience stores | 678 | 1,560 | 1,386 | 663 | 1,883 | 7,121 | 7,821 | 9,350 | 10,167 | 40,629 |
| Other stores | 2,353 | 3,206 | 2,188 | 903 | 1,949 | 6,144 | 6,534 | 7,149 | 7,403 | 37,829 |
