# Supplementary material for: Changes in the healthfulness of food and beverage purchases from 2006 to 2022 by outlet type in Mexico
Source: BMC Med. 2025 Apr 7;23:205. doi: 10.1186/s12916-025-04036-8 (PMC11974062; doi:10.1186/s12916-025-04036-8)
Supplement: Supplementary file 2 — Additional file 2. Table 2. Classification of foods from ENIGH from 2006 to 2022 according to their processing level [file 12916_2025_4036_MOESM2_ESM.docx]

**Table 2. Classification of foods from ENIGH from 2006 to 2022 according to their processing level**

| Unprocessed / minimally processed foods | A001, A003, A004, A009, A018, A019, A024, A025, A026, A027, A028, A029, A030, A031, A032, A033, A034, A035, A036, A037, A038, A039, A040, A041, A042, A043, A044, A045, A046, A057, A058, A059, A060, A061, A063, A064, A065, A066, A067, A071, A072, A073, A075, A080, A081, A093, A094, A101,  A102, A103, A104 A107, A108, A109, A110, A111, A112, A113, A114, A115,  A116, A117, A118, A119, A120, A121, A122, A123, A124, A125, A126, A127, A128, A129, A130, A131, A132, A134, A136, A137, A138, A139, A140, A141, A144, A147, A148, A149, A150, A151, A152, A153, A154, A155, A156, A157, A158, A159, A160, A161, A162, A163, A164, A165, A166, A167, A168, A169,  A170, A176, A178, A179, A199, A200, A201, A203, A204, A215, A217 |
| --- | --- |
| Culinary Ingredients | A002, A007, A077, A089, A090, A095, A096, A098, A099, A100, A173, A174, A175, A183, A184, A185, A189, A190, A191, A193, A194, A210, A222 |
| Processed foods | A005, A006, A012, A020, A047, A048, A050, A051, A068, A069, A070, A074, A083, A084, A085, A086, A087, A088, A092, A133, A135, A142, A143, A145,   A146, A171, A172, A188, A202, A224, A228, A232, A234 |
| Ultra-processed foods | A008, A010, A011, A013, A014, A015, A016, A017, A021, A022, A023, A049, A052, A053, A054, A055, A056, A062, A076, A078, A079, A082, A091, A097, A105, A106, A177, A180, A181, A182, A186, A187, A192, A195, A196, A197, A198, A205, A206, A207, A208, A209, A216, A218, A219, A220, A221, A223,  A225, A226, A227, A229, A230, A231, A233, A235, A236, A237, A238 |
