# Supplementary material for: Changes in the healthfulness of food and beverage purchases from 2006 to 2022 by outlet type in Mexico
Source: BMC Med. 2025 Apr 7;23:205. doi: 10.1186/s12916-025-04036-8 (PMC11974062; doi:10.1186/s12916-025-04036-8)
Supplement: Supplementary file 3 — Additional file 3. Table 3. Trends in the proportion of food and beverage purchases by processing level by type of food outlets [file 12916_2025_4036_MOESM3_ESM.docx]

**Table 3. Trends in the proportion of food and beverage purchases by processing level by type of food outlets (2006 - 2022)**

| Food outlets | Year | **Processing Level** | | | | | | | |
| --- | --- | --- | --- | --- | --- | --- | --- | --- | --- |
|  |  | MINIMALLY PROCESSED FOODS | | CULINARY INGREDIENTS | | PROCESSED FOODS | | ULTRA-PROCESSED FOODS | |
|  |  | **Mean** | SE | **Mean** | SE | **Mean** | SE | **Mean** | SE |
| Street vendors | 2006 | **69.5** | 0.9 | **0.6** | 0.1 | **18.1** | 0.6 | **11.8** | 0.6 |
|  | 2008 | **68.8** | 0.6 | **0.5** | 0.1 | **17.9** | 0.4 | **12.8** | 0.4 |
|  | 2010 | **66.5** | 0.8 | **0.7** | 0.1 | **19.3** | 0.6 | **13.6** | 0.5 |
|  | 2012 | **64.7** | 1.2 | **0.5** | 0.1 | **18.9** | 0.8 | **15.9** | 0.9 |
|  | 2014 | **63.9** | 0.9 | **0.8** | 0.1 | **20.5** | 0.7 | **14.7** | 0.5 |
|  | 2016 | **61.8** | 0.5 | **0.5** | 0.1 | **23.8** | 0.4 | **13.9** | 0.3 |
|  | 2018 | **62.8** | 0.5 | **0.5** | 0.0 | **23.2** | 0.4 | **13.6** | 0.3 |
|  | 2020 | **63.4** | 0.4 | **0.6** | 0.0 | **22.6** | 0.4 | **13.4** | 0.3 |
|  | 2022 | **61.6** | 0.4 | **0.6** | 0.0 | **24.9** | 0.4 | **12.9** | 0.3 |
| Street markets | 2006 | **83.3** | 0.7 | **2.8** | 0.2 | **8.8** | 0.5 | **5.0** | 0.4 |
|  | 2008 | **81.6** | 0.6 | **3.5** | 0.2 | **10.4** | 0.5 | **4.5** | 0.3 |
|  | 2010 | **81.7** | 0.6 | **3.4** | 0.3 | **9.8** | 0.5 | **5.2** | 0.3 |
|  | 2012 | **81.8** | 1.0 | **3.5** | 0.4 | **9.0** | 0.7 | **5.7** | 0.6 |
|  | 2014 | **82.0** | 0.7 | **2.3** | 0.2 | **10.8** | 0.6 | **4.8** | 0.4 |
|  | 2016 | **80.6** | 0.5 | **2.6** | 0.1 | **12.5** | 0.4 | **4.3** | 0.2 |
|  | 2018 | **80.8** | 0.5 | **2.3** | 0.1 | **12.1** | 0.4 | **4.8** | 0.2 |
|  | 2020 | **83.0** | 0.4 | **2.3** | 0.1 | **10.5** | 0.3 | **4.2** | 0.2 |
|  | 2022 | **81.0** | 0.4 | **2.5** | 0.1 | **11.6** | 0.4 | **4.8** | 0.2 |
| Acquaintances | 2006 | **-** | - | **-** | - | **-** | - | **-** | - |
|  | 2008 | **-** | - | **-** | - | **-** | - | **-** | - |
|  | 2010 | **56.0** | 1.3 | **2.3** | 0.3 | **31.3** | 1.2 | **10.4** | 0.6 |
|  | 2012 | **52.7** | 1.8 | **4.1** | 0.6 | **31.6** | 1.8 | **11.6** | 1.1 |
|  | 2014 | **55.0** | 1.3 | **3.2** | 0.5 | **30.5** | 1.1 | **11.3** | 0.7 |
|  | 2016 | **52.2** | 0.7 | **4.2** | 0.4 | **33.0** | 0.7 | **10.6** | 0.4 |
|  | 2018 | **52.1** | 0.7 | **4.0** | 0.3 | **33.6** | 0.7 | **10.2** | 0.4 |
|  | 2020 | **51.3** | 0.6 | **2.9** | 0.2 | **35.4** | 0.5 | **10.4** | 0.3 |
|  | 2022 | **51.2** | 0.6 | **2.9** | 0.2 | **36.1** | 0.5 | **9.7** | 0.3 |
| Public markets | 2006 | **80.6** | 0.5 | **2.7** | 0.2 | **9.9** | 0.4 | **6.8** | 0.3 |
|  | 2008 | **79.0** | 0.4 | **2.9** | 0.1 | **11.1** | 0.3 | **7.0** | 0.2 |
|  | 2010 | **80.2** | 0.4 | **2.8** | 0.1 | **10.0** | 0.3 | **7.0** | 0.3 |
|  | 2012 | **78.8** | 0.8 | **2.5** | 0.2 | **10.5** | 0.6 | **8.2** | 0.5 |
|  | 2014 | **79.0** | 0.5 | **2.6** | 0.2 | **11.6** | 0.4 | **6.8** | 0.3 |
|  | 2016 | **79.1** | 0.3 | **2.4** | 0.1 | **11.3** | 0.3 | **7.1** | 0.2 |
|  | 2018 | **78.8** | 0.3 | **2.6** | 0.1 | **11.5** | 0.3 | **7.0** | 0.2 |
|  | 2020 | **79.0** | 0.3 | **2.7** | 0.1 | **11.2** | 0.2 | **7.1** | 0.2 |
|  | 2022 | **78.4** | 0.3 | **2.7** | 0.1 | **11.6** | 0.2 | **7.4** | 0.2 |
| Specialty stores | 2006 | **74.6** | 0.5 | **3.2** | 0.3 | **10.9** | 0.3 | **11.4** | 0.3 |
|  | 2008 | **75.2** | 0.5 | **3.6** | 0.4 | **10.0** | 0.2 | **11.2** | 0.2 |
|  | 2010 | **74.7** | 0.5 | **3.4** | 0.4 | **10.3** | 0.3 | **11.6** | 0.3 |
|  | 2012 | **72.0** | 0.8 | **3.4** | 0.5 | **12.1** | 0.5 | **12.4** | 0.5 |
|  | 2014 | **72.6** | 0.5 | **3.5** | 0.4 | **11.6** | 0.3 | **12.3** | 0.3 |
|  | 2016 | **73.1** | 0.3 | **2.7** | 0.2 | **12.0** | 0.2 | **12.3** | 0.2 |
|  | 2018 | **72.9** | 0.3 | **2.3** | 0.1 | **13.0** | 0.2 | **11.8** | 0.2 |
|  | 2020 | **72.5** | 0.2 | **2.1** | 0.1 | **13.9** | 0.2 | **11.5** | 0.1 |
|  | 2022 | **72.1** | 0.2 | **1.8** | 0.1 | **14.2** | 0.1 | **11.9** | 0.1 |
| Smalll neighborhood stores | 2006 | **45.9** | 0.4 | **6.9** | 0.2 | **10.0** | 0.2 | **37.3** | 0.4 |
|  | 2008 | **48.2** | 0.3 | **7.2** | 0.2 | **10.4** | 0.2 | **34.1** | 0.3 |
|  | 2010 | **48.4** | 0.3 | **6.4** | 0.1 | **10.6** | 0.2 | **34.5** | 0.3 |
|  | 2012 | **46.7** | 0.5 | **6.2** | 0.2 | **10.8** | 0.3 | **36.3** | 0.5 |
|  | 2014 | **47.8** | 0.4 | **6.0** | 0.2 | **11.6** | 0.2 | **34.6** | 0.4 |
|  | 2016 | **46.9** | 0.2 | **6.3** | 0.1 | **11.6** | 0.1 | **35.1** | 0.2 |
|  | 2018 | **47.5** | 0.2 | **6.5** | 0.1 | **12.0** | 0.1 | **34.1** | 0.2 |
|  | 2020 | **48.4** | 0.2 | **6.6** | 0.1 | **11.2** | 0.1 | **33.8** | 0.2 |
|  | 2022 | **47.3** | 0.2 | **6.6** | 0.1 | **12.0** | 0.1 | **34.2** | 0.2 |
| Supermarkets | 2006 | **48.6** | 0.7 | **6.0** | 0.3 | **10.1** | 0.3 | **35.3** | 0.6 |
|  | 2008 | **50.8** | 0.5 | **6.7** | 0.2 | **10.6** | 0.3 | **31.9** | 0.4 |
|  | 2010 | **50.9** | 0.5 | **6.5** | 0.3 | **10.9** | 0.3 | **31.8** | 0.5 |
|  | 2012 | **50.6** | 0.8 | **5.5** | 0.3 | **10.7** | 0.5 | **33.1** | 0.8 |
|  | 2014 | **52.4** | 0.5 | **6.2** | 0.2 | **11.4** | 0.3 | **30.0** | 0.5 |
|  | 2016 | **51.5** | 0.3 | **6.4** | 0.1 | **11.3** | 0.2 | **30.8** | 0.3 |
|  | 2018 | **53.7** | 0.3 | **6.2** | 0.1 | **11.1** | 0.2 | **28.9** | 0.3 |
|  | 2020 | **56.0** | 0.3 | **6.7** | 0.1 | **10.6** | 0.2 | **26.7** | 0.3 |
|  | 2022 | **53.8** | 0.3 | **7.9** | 0.2 | **11.0** | 0.2 | **27.2** | 0.3 |
| Chain convenience stores | 2006 | **35.9** | 2.0 | **2.2** | 0.7 | **13.3** | 1.3 | **48.6** | 2.0 |
|  | 2008 | **32.1** | 1.8 | **1.9** | 0.4 | **18.4** | 2.1 | **47.5** | 2.0 |
|  | 2010 | **37.9** | 1.6 | **2.5** | 0.4 | **12.2** | 1.0 | **47.4** | 1.6 |
|  | 2012 | **33.1** | 1.9 | **2.4** | 0.6 | **13.8** | 1.4 | **50.7** | 2.0 |
|  | 2014 | **32.8** | 1.2 | **2.8** | 0.5 | **13.7** | 0.8 | **50.7** | 1.2 |
|  | 2016 | **32.6** | 0.6 | **3.1** | 0.2 | **12.9** | 0.5 | **51.4** | 0.7 |
|  | 2018 | **32.5** | 0.6 | **3.0** | 0.2 | **14.6** | 0.5 | **49.9** | 0.7 |
|  | 2020 | **32.2** | 0.6 | **3.8** | 0.2 | **14.5** | 0.4 | **49.5** | 0.6 |
|  | 2022 | **29.7** | 0.5 | **4.4** | 0.3 | **14.1** | 0.4 | **51.9** | 0.6 |
| Other stores | 2006 | **51.6** | 1.7 | **2.7** | 0.4 | **28.1** | 1.5 | **17.7** | 1.2 |
|  | 2008 | **48.9** | 1.6 | **3.4** | 0.8 | **30.0** | 1.5 | **17.7** | 1.1 |
|  | 2010 | **54.5** | 1.3 | **16.9** | 1.0 | **7.1** | 0.6 | **21.5** | 1.2 |
|  | 2012 | **52.6** | 2.4 | **16.9** | 1.5 | **6.1** | 0.8 | **24.4** | 1.7 |
|  | 2014 | **58.2** | 1.4 | **12.3** | 0.9 | **6.9** | 0.7 | **22.6** | 1.1 |
|  | 2016 | **54.5** | 0.9 | **13.2** | 0.5 | **8.7** | 0.5 | **23.5** | 0.7 |
|  | 2018 | **56.4** | 0.9 | **12.5** | 0.6 | **9.7** | 0.5 | **21.5** | 0.7 |
|  | 2020 | **59.7** | 0.8 | **12.5** | 0.5 | **9.1** | 0.5 | **18.7** | 0.6 |
|  | 2022 | **53.7** | 0.7 | **13.2** | 0.5 | **10.9** | 0.5 | **22.2** | 0.6 |
