# Supplementary material for: Changes in the healthfulness of food and beverage purchases from 2006 to 2022 by outlet type in Mexico
Source: BMC Med. 2025 Apr 7;23:205. doi: 10.1186/s12916-025-04036-8 (PMC11974062; doi:10.1186/s12916-025-04036-8)
Supplement: Supplementary file 4 — Additional file 4. Table 4.1 Trends in the proportion of food and beverage purchases by processing level in informal outlets stratified by education level of the head of the household, Data from ENIGH 2006 to 2022. Table 4.2. Trends in the proportion of food and beverage purchases by processing level in traditional outlets stratified by education level of the head of the household, Data from ENIGH 2006 to 2022. Table 4.3. Trends in the proportion of food and beverage purchases by processing level in supermarkets, chain convenience stores and other outlets, stratified by education level of the head of the household, Data from ENIGH 2006 to 2022 [file 12916_2025_4036_MOESM4_ESM.docx]

**Table 4.1. Trends in the proportion of food and beverage purchases by processing level in informal outlets stratified by education level of the head of the household, Data from ENIGH 2006 to 2022.**

| Food outlet | | Street vendors | | | | | | | | Street markets | | | | | | | | Acquaintances | | | | | | | |
| --- | --- | --- | --- | --- | --- | --- | --- | --- | --- | --- | --- | --- | --- | --- | --- | --- | --- | --- | --- | --- | --- | --- | --- | --- | --- |
| Processing Level | | Minimally Processed Foods | | Culinary Ingredients | | Processed foods | | Ultra-processed foods | | Minimally Processed Foods | | Culinary Ingredients | | Processed foods | | Ultra-processed foods | | Minimally Processed Foods | | Culinary Ingredients | | Processed foods | | Ultra-processed foods | |
| Education level | Year | **Mean** | SE | **Mean** | SE | **Mean** | SE | **Mean** | SE | **Mean** | SE | **Mean** | SE | **Mean** | SE | **Mean** | SE | **Mean** | SE | **Mean** | SE | **Mean** | SE | **Mean** | SE |
| Without formal education | 2006 | **75.4** | 1.8 | **0.9** | 0.3 | **11.9** | 1.4 | **11.8** | 1.4 | **85.9** | 1.6 | **3.0** | 0.6 | **7.1** | 1.2 | **4.0** | 0.7 | **-** | - | **-** | - | **-** | - | **-** | - |
|  | 2008 | **71.5** | 1.5 | **1.1** | 0.3 | **15.3** | 1.1 | **12.1** | 1.0 | **83.0** | 1.5 | **5.0** | 0.8 | **6.6** | 0.8 | **5.5** | 1.1 | **-** | - | **-** | - | **-** | - | **-** | - |
|  | 2010 | **72.0** | 2.1 | **1.4** | 0.5 | **12.7** | 1.2 | **14.0** | 1.8 | **81.6** | 1.8 | **5.7** | 0.8 | **8.7** | 1.4 | **4.0** | 1.1 | **67.3** | 2.5 | **5.5** | 1.3 | **17.5** | 1.8 | **9.7** | 1.7 |
|  | 2012 | **74.1** | 2.1 | **0.9** | 0.3 | **10.6** | 1.4 | **14.4** | 1.8 | **79.5** | 2.6 | **6.0** | 1.2 | **7.8** | 1.8 | **6.7** | 1.3 | **65.0** | 3.9 | **8.9** | 2.0 | **19.8** | 3.4 | **6.3** | 1.4 |
|  | 2014 | **68.5** | 1.9 | **1.8** | 0.6 | **16.5** | 1.6 | **13.2** | 1.3 | **85.6** | 1.9 | **3.3** | 1.1 | **7.8** | 1.4 | **3.3** | 0.9 | **63.8** | 3.2 | **7.2** | 1.7 | **19.4** | 2.3 | **9.7** | 2.0 |
|  | 2016 | **69.6** | 1.2 | **0.8** | 0.2 | **17.5** | 1.0 | **12.1** | 0.8 | **83.5** | 1.2 | **4.0** | 0.5 | **8.5** | 0.9 | **3.9** | 0.6 | **57.8** | 1.8 | **8.2** | 1.2 | **24.4** | 1.5 | **9.6** | 0.9 |
|  | 2018 | **70.3** | 1.1 | **0.7** | 0.1 | **16.7** | 0.9 | **12.3** | 0.8 | **80.4** | 1.4 | **4.0** | 0.5 | **11.0** | 1.2 | **4.6** | 0.7 | **60.7** | 1.6 | **9.2** | 1.1 | **22.8** | 1.5 | **7.4** | 1.0 |
|  | 2020 | **67.6** | 1.1 | **1.0** | 0.2 | **18.2** | 0.9 | **13.2** | 0.7 | **84.1** | 1.4 | **3.2** | 0.4 | **8.5** | 1.1 | **4.2** | 0.6 | **62.4** | 1.6 | **4.9** | 0.6 | **24.8** | 1.4 | **7.9** | 0.8 |
|  | 2022 | **70.0** | 1.2 | **0.7** | 0.1 | **17.8** | 1.0 | **11.5** | 0.8 | **84.1** | 1.2 | **3.6** | 0.5 | **8.0** | 0.9 | **4.2** | 0.6 | **60.3** | 1.6 | **6.5** | 0.8 | **25.3** | 1.4 | **8.0** | 0.8 |
| Preschool / primary school | 2006 | **70.0** | 1.1 | **0.6** | 0.2 | **17.2** | 0.7 | **12.1** | 0.7 | **82.4** | 0.9 | **3.3** | 0.3 | **9.0** | 0.6 | **5.3** | 0.6 | **-** | - | **-** | - | **-** | - | **-** | - |
|  | 2008 | **70.0** | 0.8 | **0.6** | 0.1 | **16.4** | 0.6 | **13.0** | 0.6 | **82.0** | 0.8 | **4.3** | 0.4 | **9.5** | 0.7 | **4.2** | 0.3 | **-** | - | **-** | - | **-** | - | **-** | - |
|  | 2010 | **67.8** | 1.0 | **0.8** | 0.1 | **18.6** | 0.8 | **12.8** | 0.7 | **82.0** | 0.9 | **4.0** | 0.4 | **8.9** | 0.7 | **5.2** | 0.5 | **59.2** | 1.6 | **2.2** | 0.4 | **29.2** | 1.5 | **9.4** | 0.8 |
|  | 2012 | **64.8** | 1.5 | **0.6** | 0.1 | **18.5** | 1.2 | **16.2** | 1.1 | **82.4** | 1.4 | **4.1** | 0.6 | **8.7** | 1.1 | **4.9** | 0.7 | **55.2** | 2.4 | **4.3** | 0.9 | **29.2** | 2.3 | **11.3** | 1.6 |
|  | 2014 | **68.0** | 1.1 | **0.9** | 0.2 | **17.0** | 0.8 | **14.2** | 0.7 | **82.5** | 0.9 | **2.8** | 0.4 | **9.8** | 0.7 | **4.9** | 0.5 | **57.9** | 1.7 | **3.9** | 0.8 | **27.9** | 1.4 | **10.2** | 0.9 |
|  | 2016 | **65.1** | 0.7 | **0.6** | 0.1 | **20.8** | 0.5 | **13.5** | 0.4 | **80.8** | 0.7 | **3.0** | 0.2 | **12.0** | 0.6 | **4.3** | 0.3 | **56.0** | 0.9 | **5.1** | 0.5 | **28.9** | 0.8 | **10.0** | 0.5 |
|  | 2018 | **66.3** | 0.6 | **0.6** | 0.1 | **20.2** | 0.5 | **12.9** | 0.4 | **81.8** | 0.6 | **3.1** | 0.2 | **10.6** | 0.5 | **4.5** | 0.3 | **56.7** | 1.0 | **4.8** | 0.4 | **29.4** | 0.9 | **9.1** | 0.5 |
|  | 2020 | **66.8** | 0.6 | **0.6** | 0.1 | **19.1** | 0.5 | **13.5** | 0.4 | **82.5** | 0.6 | **3.0** | 0.2 | **10.4** | 0.5 | **4.0** | 0.3 | **54.9** | 0.8 | **3.9** | 0.3 | **32.1** | 0.7 | **9.1** | 0.4 |
|  | 2022 | **66.4** | 0.6 | **0.6** | 0.1 | **20.9** | 0.5 | **12.1** | 0.4 | **81.0** | 0.6 | **3.3** | 0.3 | **11.2** | 0.6 | **4.5** | 0.3 | **55.2** | 0.8 | **3.9** | 0.3 | **32.2** | 0.7 | **8.7** | 0.4 |
| Middle school / high school | 2006 | **63.7** | 1.6 | **0.5** | 0.2 | **23.5** | 1.3 | **12.2** | 0.9 | **84.3** | 1.5 | **2.2** | 0.5 | **8.6** | 1.0 | **5.0** | 0.8 | **-** | - | **-** | - | **-** | - | **-** | - |
|  | 2008 | **67.2** | 0.9 | **0.4** | 0.1 | **20.0** | 0.7 | **12.4** | 0.5 | **80.8** | 0.9 | **2.5** | 0.3 | **12.2** | 0.8 | **4.6** | 0.4 | **-** | - | **-** | - | **-** | - | **-** | - |
|  | 2010 | **63.7** | 1.2 | **0.4** | 0.1 | **21.6** | 0.9 | **14.3** | 0.7 | **79.8** | 1.0 | **2.5** | 0.2 | **12.0** | 0.9 | **5.7** | 0.5 | **50.6** | 2.1 | **1.4** | 0.5 | **36.1** | 2.1 | **11.9** | 1.3 |
|  | 2012 | **62.6** | 1.9 | **0.3** | 0.1 | **20.0** | 1.3 | **17.1** | 1.6 | **81.3** | 1.7 | **3.0** | 0.5 | **8.8** | 1.2 | **6.8** | 1.1 | **46.7** | 2.7 | **2.6** | 0.7 | **37.6** | 2.5 | **13.1** | 1.7 |
|  | 2014 | **61.0** | 1.4 | **0.5** | 0.1 | **22.9** | 1.0 | **15.6** | 0.8 | **81.3** | 1.1 | **2.1** | 0.3 | **11.6** | 0.9 | **5.0** | 0.6 | **49.5** | 1.9 | **1.4** | 0.4 | **35.8** | 1.8 | **13.3** | 1.3 |
|  | 2016 | **58.8** | 0.7 | **0.4** | 0.1 | **26.3** | 0.6 | **14.5** | 0.5 | **79.3** | 0.8 | **2.4** | 0.2 | **13.9** | 0.7 | **4.5** | 0.3 | **48.3** | 1.0 | **2.6** | 0.3 | **37.5** | 1.0 | **11.7** | 0.6 |
|  | 2018 | **59.7** | 0.7 | **0.4** | 0.1 | **25.3** | 0.6 | **14.6** | 0.5 | **79.9** | 0.7 | **1.9** | 0.2 | **13.0** | 0.6 | **5.2** | 0.4 | **47.9** | 1.0 | **2.4** | 0.3 | **38.4** | 0.9 | **11.2** | 0.6 |
|  | 2020 | **60.6** | 0.6 | **0.5** | 0.1 | **25.4** | 0.5 | **13.6** | 0.4 | **82.6** | 0.6 | **2.0** | 0.1 | **11.1** | 0.5 | **4.3** | 0.3 | **48.0** | 0.8 | **1.8** | 0.2 | **38.9** | 0.7 | **11.3** | 0.5 |
|  | 2022 | **58.6** | 0.6 | **0.5** | 0.1 | **27.3** | 0.5 | **13.7** | 0.4 | **80.4** | 0.6 | **2.2** | 0.2 | **12.3** | 0.5 | **5.1** | 0.3 | **48.2** | 0.8 | **1.9** | 0.2 | **39.3** | 0.7 | **10.7** | 0.5 |
| Higher education | 2006 | **71.4** | 1.8 | **0.3** | 0.2 | **19.5** | 1.5 | **8.7** | 1.1 | **84.7** | 2.2 | **1.0** | 0.3 | **9.9** | 1.8 | **4.4** | 1.5 | **-** | - | **-** | - | **-** | - | **-** | - |
|  | 2008 | **64.9** | 1.7 | **0.2** | 0.1 | **21.0** | 1.3 | **13.9** | 1.2 | **81.5** | 2.5 | **1.3** | 0.3 | **12.4** | 2.4 | **4.7** | 0.8 | **-** | - | **-** | - | **-** | - | **-** | - |
|  | 2010 | **64.8** | 1.8 | **0.3** | 0.1 | **21.0** | 1.6 | **13.9** | 1.3 | **88.4** | 1.5 | **1.8** | 0.4 | **5.9** | 0.9 | **4.0** | 1.0 | **41.5** | 3.9 | **1.5** | 1.2 | **44.8** | 4.0 | **12.1** | 2.2 |
|  | 2012 | **63.0** | 3.6 | **0.8** | 0.5 | **26.1** | 3.2 | **10.1** | 1.9 | **84.1** | 3.0 | **1.1** | 0.5 | **12.0** | 2.6 | **2.7** | 1.2 | **40.6** | 6.5 | **1.1** | 1.1 | **41.4** | 6.9 | **16.9** | 5.4 |
|  | 2014 | **55.4** | 2.3 | **1.1** | 0.7 | **28.5** | 2.1 | **15.0** | 1.6 | **81.1** | 2.2 | **0.9** | 0.2 | **13.4** | 2.0 | **4.6** | 0.9 | **49.4** | 4.1 | **1.7** | 0.9 | **38.9** | 3.7 | **10.1** | 2.5 |
|  | 2016 | **55.5** | 1.3 | **0.6** | 0.2 | **29.6** | 1.3 | **14.2** | 0.9 | **83.6** | 1.3 | **1.0** | 0.2 | **11.5** | 1.2 | **3.8** | 0.6 | **43.7** | 2.3 | **2.0** | 0.7 | **44.0** | 2.2 | **10.3** | 1.3 |
|  | 2018 | **57.6** | 1.4 | **0.4** | 0.1 | **29.1** | 1.2 | **12.9** | 0.9 | **81.7** | 1.3 | **0.8** | 0.2 | **13.6** | 1.2 | **3.9** | 0.6 | **39.9** | 2.1 | **1.8** | 0.6 | **44.0** | 2.1 | **14.3** | 1.6 |
|  | 2020 | **61.2** | 1.1 | **0.5** | 0.1 | **26.0** | 1.0 | **12.2** | 0.7 | **85.9** | 1.1 | **1.1** | 0.2 | **9.2** | 0.9 | **3.8** | 0.6 | **41.0** | 1.7 | **1.4** | 0.3 | **43.2** | 1.6 | **14.3** | 1.3 |
|  | 2022 | **55.0** | 1.1 | **0.5** | 0.1 | **31.4** | 1.0 | **13.2** | 0.7 | **82.1** | 1.1 | **1.6** | 0.2 | **11.8** | 0.9 | **4.5** | 0.6 | **43.1** | 1.7 | **1.1** | 0.3 | **45.2** | 1.7 | **10.6** | 0.9 |

**Table 4.2. Trends in the proportion of food and beverage purchases by processing level in traditional outlets stratified by education level of the head of the household, Data from ENIGH 2006 to 2022.**

| Food outlet | | Public Markets | | | | | | | | Specialty stores | | | | | | | | Small neighborhood stores | | | | | | | |
| --- | --- | --- | --- | --- | --- | --- | --- | --- | --- | --- | --- | --- | --- | --- | --- | --- | --- | --- | --- | --- | --- | --- | --- | --- | --- |
| Processing Level | | Minimally Processed Foods | | Culinary Ingredients | | Processed foods | | Ultra-processed foods | | Minimally Processed Foods | | Culinary Ingredients | | Processed foods | | Ultra-processed foods | | Minimally Processed Foods | | Culinary Ingredients | | Processed foods | | Ultra-processed foods | |
| Education level | Year | **Mean** | SE | **Mean** | SE | **Mean** | SE | **Mean** | SE | **Mean** | SE | **Mean** | SE | **Mean** | SE | **Mean** | SE | **Mean** | SE | **Mean** | SE | **Mean** | SE | **Mean** | SE |
| Without formal education | 2006 | **82.1** | 1.4 | **4.2** | 0.8 | **7.2** | 0.9 | **6.5** | 1.0 | **74.9** | 1.9 | **11.5** | 1.7 | **7.0** | 0.8 | **6.5** | 0.6 | **50.0** | 0.9 | **11.0** | 0.6 | **8.1** | 0.5 | **30.9** | 0.9 |
|  | 2008 | **80.1** | 1.1 | **3.8** | 0.5 | **10.1** | 0.9 | **6.0** | 0.6 | **76.8** | 1.3 | **9.7** | 1.1 | **6.2** | 0.5 | **7.3** | 0.6 | **51.6** | 0.8 | **10.6** | 0.5 | **8.9** | 0.5 | **28.9** | 0.7 |
|  | 2010 | **79.9** | 1.4 | **4.3** | 0.7 | **9.0** | 0.8 | **6.9** | 0.8 | **75.8** | 2.5 | **12.0** | 2.6 | **5.9** | 0.5 | **6.3** | 0.5 | **53.6** | 0.9 | **9.9** | 0.4 | **9.0** | 0.5 | **27.4** | 0.8 |
|  | 2012 | **79.1** | 2.3 | **3.5** | 0.8 | **10.5** | 1.8 | **6.9** | 1.2 | **73.9** | 2.3 | **11.0** | 2.2 | **8.5** | 1.0 | **6.6** | 0.9 | **52.6** | 1.6 | **9.4** | 0.7 | **9.9** | 0.9 | **28.1** | 1.4 |
|  | 2014 | **81.8** | 1.7 | **2.8** | 0.5 | **9.1** | 1.2 | **6.2** | 0.9 | **73.4** | 1.8 | **11.2** | 1.6 | **7.4** | 0.7 | **8.0** | 0.9 | **53.5** | 1.0 | **9.1** | 0.5 | **10.1** | 0.6 | **27.4** | 1.0 |
|  | 2016 | **79.0** | 1.2 | **3.6** | 0.4 | **10.8** | 0.9 | **6.6** | 0.7 | **76.4** | 0.9 | **8.1** | 0.7 | **8.0** | 0.4 | **7.5** | 0.5 | **51.8** | 0.7 | **9.0** | 0.3 | **9.7** | 0.3 | **29.5** | 0.6 |
|  | 2018 | **80.8** | 1.0 | **3.1** | 0.3 | **9.7** | 0.7 | **6.4** | 0.6 | **77.3** | 0.8 | **7.5** | 0.7 | **8.9** | 0.5 | **6.4** | 0.4 | **52.2** | 0.6 | **10.3** | 0.3 | **9.2** | 0.3 | **28.3** | 0.5 |
|  | 2020 | **79.8** | 1.1 | **3.2** | 0.4 | **10.7** | 0.9 | **6.2** | 0.6 | **79.2** | 0.7 | **6.0** | 0.5 | **8.2** | 0.4 | **6.6** | 0.4 | **52.6** | 0.6 | **9.5** | 0.3 | **9.1** | 0.3 | **28.7** | 0.5 |
|  | 2022 | **79.6** | 1.1 | **3.1** | 0.4 | **10.8** | 1.0 | **6.5** | 0.6 | **78.5** | 0.7 | **4.8** | 0.4 | **9.1** | 0.4 | **7.6** | 0.4 | **50.8** | 0.5 | **8.9** | 0.3 | **10.1** | 0.3 | **30.1** | 0.5 |
| Preschool / primary school | 2006 | **80.6** | 0.7 | **2.8** | 0.3 | **9.5** | 0.4 | **7.0** | 0.4 | **76.6** | 0.6 | **3.3** | 0.3 | **10.0** | 0.4 | **10.1** | 0.4 | **46.9** | 0.5 | **7.4** | 0.2 | **9.6** | 0.2 | **36.0** | 0.5 |
|  | 2008 | **79.8** | 0.6 | **3.2** | 0.2 | **10.3** | 0.5 | **6.7** | 0.4 | **77.0** | 0.7 | **5.1** | 0.6 | **8.4** | 0.3 | **9.5** | 0.3 | **49.6** | 0.4 | **8.6** | 0.2 | **10.1** | 0.2 | **31.7** | 0.4 |
|  | 2010 | **80.7** | 0.6 | **3.0** | 0.2 | **9.9** | 0.5 | **6.4** | 0.3 | **76.6** | 0.6 | **4.4** | 0.5 | **9.5** | 0.4 | **9.6** | 0.3 | **50.5** | 0.4 | **7.4** | 0.2 | **10.1** | 0.2 | **32.0** | 0.4 |
|  | 2012 | **78.5** | 1.2 | **2.7** | 0.3 | **10.9** | 1.1 | **7.9** | 0.7 | **73.8** | 1.0 | **4.9** | 0.8 | **11.0** | 0.6 | **10.3** | 0.6 | **48.5** | 0.7 | **7.2** | 0.3 | **10.6** | 0.4 | **33.8** | 0.7 |
|  | 2014 | **79.2** | 0.8 | **3.3** | 0.3 | **10.8** | 0.6 | **6.7** | 0.5 | **75.7** | 0.7 | **5.3** | 0.6 | **9.6** | 0.4 | **9.5** | 0.4 | **50.0** | 0.5 | **6.9** | 0.2 | **11.0** | 0.3 | **32.2** | 0.5 |
|  | 2016 | **80.1** | 0.5 | **2.7** | 0.1 | **10.5** | 0.4 | **6.6** | 0.3 | **77.0** | 0.4 | **4.1** | 0.3 | **9.7** | 0.2 | **9.2** | 0.2 | **48.9** | 0.3 | **7.5** | 0.1 | **11.1** | 0.2 | **32.5** | 0.3 |
|  | 2018 | **79.2** | 0.5 | **3.1** | 0.2 | **11.1** | 0.4 | **6.6** | 0.3 | **76.9** | 0.4 | **3.4** | 0.2 | **10.9** | 0.2 | **8.8** | 0.2 | **49.6** | 0.3 | **7.5** | 0.1 | **11.4** | 0.2 | **31.5** | 0.3 |
|  | 2020 | **78.8** | 0.5 | **3.1** | 0.1 | **11.1** | 0.4 | **6.9** | 0.3 | **76.8** | 0.3 | **3.4** | 0.2 | **11.1** | 0.2 | **8.8** | 0.2 | **50.4** | 0.3 | **7.6** | 0.1 | **10.7** | 0.1 | **31.3** | 0.3 |
|  | 2022 | **78.0** | 0.5 | **3.3** | 0.2 | **11.5** | 0.4 | **7.2** | 0.3 | **76.2** | 0.3 | **2.9** | 0.2 | **11.8** | 0.2 | **9.1** | 0.2 | **49.4** | 0.3 | **7.8** | 0.1 | **11.2** | 0.1 | **31.6** | 0.3 |
| Middle school / high school | 2006 | **79.1** | 0.9 | **2.1** | 0.2 | **12.2** | 0.8 | **6.6** | 0.5 | **73.9** | 0.9 | **0.6** | 0.1 | **12.5** | 0.6 | **13.0** | 0.6 | **42.9** | 0.7 | **4.9** | 0.4 | **11.4** | 0.4 | **40.8** | 0.6 |
|  | 2008 | **77.1** | 0.7 | **2.5** | 0.2 | **12.4** | 0.5 | **8.0** | 0.4 | **74.8** | 0.5 | **1.2** | 0.1 | **11.5** | 0.4 | **12.5** | 0.4 | **46.4** | 0.4 | **5.4** | 0.2 | **11.1** | 0.2 | **37.1** | 0.4 |
|  | 2010 | **79.7** | 0.7 | **2.5** | 0.2 | **9.9** | 0.4 | **7.8** | 0.4 | **74.0** | 0.6 | **1.3** | 0.2 | **11.4** | 0.4 | **13.4** | 0.4 | **45.9** | 0.4 | **5.0** | 0.1 | **11.4** | 0.2 | **37.7** | 0.4 |
|  | 2012 | **77.3** | 1.2 | **2.4** | 0.4 | **11.4** | 1.0 | **8.8** | 0.7 | **72.2** | 1.0 | **1.2** | 0.2 | **12.8** | 0.7 | **13.8** | 0.7 | **44.3** | 0.7 | **5.0** | 0.3 | **11.4** | 0.4 | **39.4** | 0.7 |
|  | 2014 | **78.3** | 0.8 | **2.0** | 0.2 | **12.6** | 0.6 | **7.2** | 0.4 | **72.4** | 0.6 | **1.4** | 0.2 | **12.6** | 0.4 | **13.5** | 0.4 | **45.8** | 0.5 | **5.0** | 0.2 | **12.3** | 0.3 | **36.8** | 0.4 |
|  | 2016 | **78.2** | 0.5 | **2.3** | 0.1 | **12.0** | 0.4 | **7.5** | 0.3 | **72.1** | 0.3 | **1.3** | 0.1 | **13.0** | 0.2 | **13.6** | 0.3 | **45.3** | 0.3 | **5.4** | 0.1 | **12.0** | 0.2 | **37.3** | 0.3 |
|  | 2018 | **78.2** | 0.5 | **2.5** | 0.1 | **11.9** | 0.4 | **7.3** | 0.3 | **72.2** | 0.3 | **1.3** | 0.1 | **13.7** | 0.2 | **12.7** | 0.2 | **45.9** | 0.3 | **5.6** | 0.1 | **12.3** | 0.2 | **36.2** | 0.3 |
|  | 2020 | **78.5** | 0.4 | **2.6** | 0.1 | **11.5** | 0.3 | **7.4** | 0.3 | **71.7** | 0.3 | **1.2** | 0.1 | **14.4** | 0.2 | **12.6** | 0.2 | **47.0** | 0.2 | **5.9** | 0.1 | **11.6** | 0.1 | **35.5** | 0.2 |
|  | 2022 | **77.9** | 0.4 | **2.5** | 0.1 | **11.7** | 0.3 | **7.9** | 0.2 | **71.6** | 0.3 | **1.1** | 0.1 | **14.6** | 0.2 | **12.6** | 0.2 | **46.0** | 0.2 | **5.9** | 0.1 | **12.3** | 0.1 | **35.9** | 0.2 |
| Higher education | 2006 | **81.8** | 1.2 | **1.4** | 0.2 | **9.9** | 0.9 | **6.9** | 0.8 | **63.6** | 1.3 | **0.4** | 0.1 | **16.1** | 1.0 | **19.9** | 1.1 | **41.8** | 1.0 | **3.4** | 0.3 | **10.7** | 0.6 | **44.1** | 1.0 |
|  | 2008 | **80.9** | 1.1 | **2.0** | 0.3 | **11.2** | 0.8 | **5.9** | 0.5 | **67.7** | 1.0 | **0.6** | 0.1 | **14.9** | 0.8 | **16.8** | 0.7 | **44.2** | 0.9 | **4.0** | 0.3 | **10.9** | 0.5 | **40.9** | 0.8 |
|  | 2010 | **79.5** | 1.0 | **2.1** | 0.4 | **11.2** | 0.8 | **7.1** | 0.6 | **69.8** | 1.1 | **0.4** | 0.0 | **12.9** | 0.7 | **17.0** | 0.8 | **43.2** | 0.9 | **4.2** | 0.3 | **11.7** | 0.5 | **40.8** | 0.9 |
|  | 2012 | **86.2** | 1.5 | **1.1** | 0.3 | **5.4** | 0.9 | **7.3** | 1.1 | **63.8** | 2.1 | **0.7** | 0.2 | **16.7** | 1.8 | **18.8** | 1.6 | **43.2** | 2.2 | **3.3** | 0.5 | **10.2** | 0.8 | **43.4** | 2.1 |
|  | 2014 | **79.4** | 1.4 | **2.1** | 0.4 | **12.5** | 1.1 | **6.1** | 0.7 | **63.8** | 1.2 | **0.4** | 0.1 | **16.9** | 0.9 | **19.0** | 1.0 | **43.3** | 1.1 | **4.1** | 0.5 | **12.1** | 0.7 | **40.4** | 1.1 |
|  | 2016 | **79.6** | 0.9 | **1.5** | 0.2 | **11.3** | 0.7 | **7.7** | 0.6 | **63.3** | 0.7 | **0.5** | 0.1 | **17.0** | 0.5 | **19.2** | 0.6 | **43.2** | 0.6 | **4.0** | 0.2 | **13.4** | 0.4 | **39.4** | 0.6 |
|  | 2018 | **78.9** | 0.8 | **1.8** | 0.2 | **12.0** | 0.7 | **7.3** | 0.5 | **63.8** | 0.7 | **0.5** | 0.1 | **17.2** | 0.5 | **18.5** | 0.6 | **44.3** | 0.6 | **4.6** | 0.2 | **13.8** | 0.4 | **37.3** | 0.6 |
|  | 2020 | **80.3** | 0.7 | **1.8** | 0.1 | **10.8** | 0.6 | **7.0** | 0.4 | **62.3** | 0.6 | **0.5** | 0.0 | **20.7** | 0.5 | **16.5** | 0.5 | **45.3** | 0.6 | **4.5** | 0.2 | **12.7** | 0.3 | **37.6** | 0.6 |
|  | 2022 | **80.4** | 0.7 | **1.8** | 0.2 | **11.4** | 0.6 | **6.5** | 0.4 | **63.0** | 0.5 | **0.6** | 0.1 | **19.5** | 0.4 | **17.0** | 0.4 | **44.8** | 0.5 | **4.9** | 0.2 | **13.4** | 0.3 | **37.0** | 0.5 |

**Table 4.3. Trends in the proportion of food and beverage purchases by processing level in supermarkets, chain convenience stores and other outlets, stratified by education level of the head of the household, Data from ENIGH 2006 to 2022.**

| Food outlet | | Supermarkets | | | | | | | | Chain convenience stores | | | | | | | | Other stores | | | | | | | |
| --- | --- | --- | --- | --- | --- | --- | --- | --- | --- | --- | --- | --- | --- | --- | --- | --- | --- | --- | --- | --- | --- | --- | --- | --- | --- |
| Processing Level | | Minimally Processed Foods | | Culinary Ingredients | | Processed foods | | Ultra-processed foods | | Minimally Processed Foods | | Culinary Ingredients | | Processed foods | | Ultra-processed foods | | Minimally Processed Foods | | Culinary Ingredients | | Processed foods | | Ultra-processed foods | |
| Education level | Year | **Mean** | SE | **Mean** | SE | **Mean** | SE | **Mean** | SE | **Mean** | SE | **Mean** | SE | **Mean** | SE | **Mean** | SE | **Mean** | SE | **Mean** | SE | **Mean** | SE | **Mean** | SE |
| Without formal education | 2006 | **51.2** | 3.4 | **13.9** | 2.5 | **6.0** | 1.2 | **28.8** | 2.8 | **52.8** | 18.2 | **1.1** | 1.1 | **0.2** | 0.2 | **46.0** | 18.4 | **57.0** | 3.6 | **5.1** | 1.5 | **26.8** | 4.1 | **11.0** | 3.3 |
|  | 2008 | **54.9** | 2.2 | **9.7** | 1.3 | **7.7** | 1.1 | **27.7** | 2.0 | **35.6** | 9.7 | **2.0** | 1.8 | **32.0** | 14.8 | **30.5** | 9.4 | **61.9** | 3.8 | **4.0** | 1.3 | **22.9** | 3.4 | **11.3** | 2.2 |
|  | 2010 | **55.7** | 3.1 | **8.1** | 1.0 | **9.5** | 1.4 | **26.7** | 3.0 | **36.9** | 9.1 | **7.6** | 4.4 | **16.9** | 10.6 | **38.6** | 9.6 | **59.5** | 2.2 | **22.4** | 2.5 | **5.2** | 1.4 | **13.0** | 2.0 |
|  | 2012 | **47.6** | 4.2 | **12.1** | 3.3 | **8.0** | 2.2 | **32.4** | 3.3 | **26.2** | 7.3 | **2.1** | 1.6 | **33.8** | 10.3 | **37.9** | 9.1 | **53.1** | 4.3 | **21.8** | 3.6 | **5.0** | 1.4 | **20.2** | 3.8 |
|  | 2014 | **57.3** | 3.1 | **11.3** | 2.0 | **9.3** | 1.6 | **22.1** | 2.6 | **41.3** | 10.3 | **6.5** | 3.4 | **9.4** | 4.3 | **42.8** | 9.6 | **58.6** | 3.4 | **17.8** | 2.5 | **2.7** | 0.8 | **20.9** | 2.9 |
|  | 2016 | **51.1** | 1.8 | **11.0** | 0.9 | **10.7** | 1.3 | **27.3** | 1.6 | **34.3** | 4.2 | **6.1** | 3.0 | **12.8** | 4.8 | **46.9** | 4.1 | **60.6** | 2.8 | **18.7** | 1.8 | **5.5** | 1.2 | **15.3** | 1.9 |
|  | 2018 | **53.4** | 1.8 | **10.7** | 0.9 | **11.5** | 1.4 | **24.3** | 1.5 | **43.5** | 5.4 | **2.7** | 1.1 | **13.1** | 3.4 | **40.8** | 4.7 | **57.9** | 2.0 | **20.6** | 1.6 | **5.7** | 1.0 | **15.7** | 1.4 |
|  | 2020 | **57.5** | 1.9 | **10.1** | 1.1 | **8.2** | 0.9 | **24.2** | 1.7 | **37.0** | 4.0 | **7.7** | 1.9 | **12.3** | 2.4 | **43.0** | 4.1 | **61.7** | 1.9 | **19.6** | 1.4 | **4.4** | 0.8 | **14.2** | 1.2 |
|  | 2022 | **51.7** | 2.0 | **13.1** | 1.3 | **10.2** | 1.4 | **25.0** | 1.7 | **32.3** | 3.7 | **15.1** | 3.5 | **19.9** | 4.2 | **32.7** | 4.2 | **58.3** | 2.0 | **23.2** | 1.6 | **5.1** | 0.8 | **13.3** | 1.4 |
| Preschool / primary school | 2006 | **48.6** | 1.0 | **7.0** | 0.5 | **9.5** | 0.5 | **34.9** | 0.9 | **37.9** | 3.6 | **2.9** | 2.0 | **15.0** | 2.8 | **44.2** | 3.5 | **56.0** | 2.1 | **2.9** | 0.5 | **26.9** | 1.9 | **14.3** | 1.2 |
|  | 2008 | **51.4** | 0.9 | **8.6** | 0.5 | **10.6** | 0.5 | **29.3** | 0.8 | **31.4** | 3.0 | **3.6** | 1.4 | **18.9** | 2.8 | **46.1** | 3.9 | **54.6** | 2.8 | **3.7** | 1.4 | **26.5** | 2.1 | **15.1** | 1.8 |
|  | 2010 | **51.6** | 0.9 | **8.5** | 0.6 | **10.5** | 0.5 | **29.4** | 0.9 | **39.7** | 3.8 | **3.8** | 1.1 | **13.7** | 2.4 | **42.8** | 3.3 | **55.3** | 1.6 | **20.6** | 1.4 | **5.6** | 0.6 | **18.5** | 1.3 |
|  | 2012 | **51.2** | 1.5 | **6.2** | 0.6 | **10.5** | 1.0 | **32.1** | 1.4 | **30.9** | 3.6 | **3.5** | 1.6 | **13.4** | 2.8 | **52.3** | 3.6 | **55.3** | 2.5 | **20.2** | 1.8 | **5.2** | 0.8 | **19.3** | 1.7 |
|  | 2014 | **53.4** | 1.0 | **7.4** | 0.4 | **9.8** | 0.5 | **29.4** | 0.9 | **35.8** | 2.5 | **4.8** | 1.3 | **13.2** | 2.3 | **46.2** | 2.6 | **59.3** | 1.8 | **13.3** | 1.2 | **5.8** | 0.9 | **21.6** | 1.4 |
|  | 2016 | **53.4** | 0.6 | **8.4** | 0.3 | **10.1** | 0.4 | **28.2** | 0.5 | **32.6** | 1.4 | **3.9** | 0.4 | **13.0** | 1.1 | **50.5** | 1.5 | **56.8** | 1.2 | **17.1** | 0.9 | **6.2** | 0.5 | **20.0** | 0.9 |
|  | 2018 | **55.5** | 0.6 | **8.2** | 0.3 | **9.2** | 0.3 | **27.0** | 0.5 | **32.2** | 1.3 | **5.5** | 0.6 | **16.5** | 1.1 | **45.8** | 1.4 | **58.7** | 1.1 | **15.5** | 0.8 | **7.8** | 0.6 | **18.0** | 0.8 |
|  | 2020 | **56.8** | 0.6 | **8.4** | 0.3 | **9.8** | 0.4 | **25.0** | 0.5 | **34.7** | 1.2 | **5.3** | 0.5 | **14.3** | 0.9 | **45.7** | 1.2 | **62.5** | 1.1 | **15.7** | 0.8 | **6.6** | 0.6 | **15.2** | 0.7 |
|  | 2022 | **53.5** | 0.6 | **10.0** | 0.4 | **10.5** | 0.4 | **25.9** | 0.5 | **32.6** | 1.2 | **5.8** | 0.6 | **14.4** | 1.0 | **47.1** | 1.3 | **56.5** | 1.2 | **17.2** | 0.9 | **7.1** | 0.6 | **19.1** | 0.9 |
| Middle school / high school | 2006 | **46.6** | 1.1 | **5.0** | 0.3 | **10.7** | 0.6 | **37.7** | 1.1 | **37.0** | 3.3 | **2.6** | 1.0 | **11.4** | 1.8 | **49.0** | 3.1 | **40.9** | 3.0 | **1.2** | 0.4 | **34.6** | 2.8 | **23.4** | 2.4 |
|  | 2008 | **49.5** | 0.7 | **6.2** | 0.3 | **10.6** | 0.4 | **33.7** | 0.7 | **29.2** | 2.6 | **1.2** | 0.5 | **19.1** | 3.4 | **50.5** | 3.2 | **41.9** | 2.2 | **2.1** | 0.5 | **35.8** | 2.6 | **20.3** | 1.7 |
|  | 2010 | **49.4** | 0.8 | **6.3** | 0.3 | **10.5** | 0.5 | **33.8** | 0.7 | **36.6** | 2.3 | **2.5** | 0.7 | **12.6** | 1.7 | **48.3** | 2.2 | **52.1** | 2.8 | **10.5** | 1.3 | **8.1** | 1.2 | **29.3** | 2.4 |
|  | 2012 | **49.2** | 1.3 | **5.5** | 0.5 | **11.1** | 0.8 | **34.2** | 1.2 | **28.9** | 2.5 | **3.0** | 0.9 | **14.5** | 2.0 | **53.7** | 2.9 | **49.5** | 4.9 | **11.1** | 2.0 | **6.7** | 1.7 | **32.7** | 4.1 |
|  | 2014 | **51.6** | 0.7 | **6.5** | 0.3 | **11.3** | 0.4 | **30.6** | 0.7 | **31.0** | 1.5 | **2.6** | 0.5 | **14.3** | 1.1 | **52.2** | 1.7 | **59.1** | 2.5 | **11.3** | 1.3 | **8.5** | 1.1 | **21.1** | 1.8 |
|  | 2016 | **50.0** | 0.5 | **6.1** | 0.2 | **11.2** | 0.3 | **32.7** | 0.4 | **32.1** | 0.8 | **3.1** | 0.3 | **12.1** | 0.6 | **52.7** | 0.9 | **54.8** | 1.4 | **9.9** | 0.6 | **8.9** | 0.9 | **26.4** | 1.2 |
|  | 2018 | **53.1** | 0.5 | **5.9** | 0.2 | **11.0** | 0.3 | **30.0** | 0.4 | **32.6** | 0.8 | **2.9** | 0.3 | **14.2** | 0.6 | **50.3** | 0.9 | **57.6** | 1.4 | **8.9** | 0.6 | **10.1** | 0.9 | **23.4** | 1.1 |
|  | 2020 | **55.4** | 0.5 | **6.6** | 0.2 | **10.4** | 0.3 | **27.6** | 0.4 | **31.4** | 0.8 | **3.9** | 0.3 | **14.8** | 0.6 | **49.9** | 0.8 | **60.4** | 1.3 | **10.1** | 0.7 | **9.3** | 0.8 | **20.2** | 1.1 |
|  | 2022 | **53.3** | 0.4 | **8.1** | 0.2 | **10.9** | 0.2 | **27.7** | 0.4 | **28.8** | 0.7 | **4.5** | 0.4 | **14.2** | 0.6 | **52.5** | 0.8 | **54.0** | 1.2 | **10.6** | 0.6 | **10.9** | 0.8 | **24.5** | 1.0 |
| Higher education | 2006 | **50.6** | 1.0 | **4.2** | 0.3 | **11.2** | 0.6 | **34.0** | 0.9 | **32.7** | 3.1 | **1.2** | 0.5 | **14.2** | 2.1 | **51.9** | 3.3 | **41.2** | 3.7 | **1.7** | 0.5 | **24.3** | 3.1 | **32.9** | 3.2 |
|  | 2008 | **51.7** | 0.9 | **4.2** | 0.3 | **11.0** | 0.5 | **33.1** | 0.8 | **37.1** | 2.9 | **1.6** | 0.4 | **16.4** | 2.4 | **44.9** | 2.6 | **36.8** | 3.0 | **5.7** | 2.1 | **32.3** | 3.1 | **25.2** | 2.6 |
|  | 2010 | **52.0** | 0.9 | **3.8** | 0.3 | **12.3** | 0.5 | **31.9** | 0.8 | **38.5** | 2.8 | **1.3** | 0.3 | **10.7** | 1.2 | **49.4** | 2.5 | **48.6** | 4.2 | **4.3** | 1.3 | **15.7** | 3.5 | **31.4** | 3.9 |
|  | 2012 | **53.2** | 1.6 | **3.7** | 0.4 | **10.7** | 0.8 | **32.4** | 1.5 | **42.8** | 4.0 | **0.4** | 0.3 | **11.5** | 2.4 | **45.2** | 3.6 | **47.6** | 6.7 | **11.6** | 3.9 | **10.6** | 2.8 | **30.1** | 5.2 |
|  | 2014 | **52.5** | 1.1 | **3.7** | 0.3 | **13.6** | 0.7 | **30.2** | 0.9 | **33.0** | 2.1 | **1.3** | 0.3 | **13.6** | 1.5 | **52.1** | 2.0 | **49.9** | 3.9 | **4.6** | 1.4 | **11.2** | 2.2 | **34.3** | 3.3 |
|  | 2016 | **52.9** | 0.7 | **4.1** | 0.2 | **12.7** | 0.4 | **30.3** | 0.6 | **33.5** | 1.2 | **2.3** | 0.5 | **14.0** | 1.0 | **50.1** | 1.3 | **41.4** | 2.5 | **5.0** | 1.0 | **19.4** | 1.9 | **34.3** | 2.4 |
|  | 2018 | **53.3** | 0.7 | **4.4** | 0.2 | **13.1** | 0.5 | **29.2** | 0.6 | **32.0** | 1.2 | **1.6** | 0.2 | **14.1** | 0.9 | **52.3** | 1.2 | **44.4** | 2.9 | **6.0** | 1.6 | **17.6** | 2.1 | **32.0** | 2.6 |
|  | 2020 | **56.4** | 0.7 | **5.1** | 0.2 | **11.8** | 0.4 | **26.8** | 0.6 | **31.6** | 1.1 | **2.3** | 0.3 | **14.1** | 0.8 | **52.0** | 1.1 | **48.5** | 2.1 | **5.7** | 0.8 | **18.5** | 1.7 | **27.3** | 1.9 |
|  | 2022 | **55.4** | 0.6 | **5.3** | 0.2 | **11.9** | 0.3 | **27.5** | 0.5 | **29.1** | 1.0 | **2.7** | 0.3 | **13.4** | 0.8 | **54.8** | 1.0 | **44.4** | 1.8 | **4.9** | 0.7 | **22.2** | 1.7 | **28.5** | 1.7 |
