# Supplementary material for: Changes in the healthfulness of food and beverage purchases from 2006 to 2022 by outlet type in Mexico
Source: BMC Med. 2025 Apr 7;23:205. doi: 10.1186/s12916-025-04036-8 (PMC11974062; doi:10.1186/s12916-025-04036-8)
Supplement: Supplementary file 5 — Additional file 5. Table 5.1 Trends in the proportion of food and beverage purchases by processing level in informal outlets stratified by urbanicity, Data from ENIGH 2006 to 2022. Table 5.2. Trends in the proportion of food and beverage purchases by processing level in traditional outlets stratified by urbanicity, Data from ENIGH 2006 to 2022. Table 5.3. Trends in the proportion of food and beverage purchases by processing level in supermarkets, chain convenience stores and other outlets, stratified by urbanicity, Data from ENIGH 2006 to 2022 [file 12916_2025_4036_MOESM5_ESM.docx]

**Table 5.1. Trends in the proportion of food and beverage purchases by processing level in informal outlets stratified by urbanicity, Data from ENIGH 2006 to 2022.**

| Food outlet | | Street vendors | | | | | | | | Street markets | | | | | | | | Acquaintances | | | | | | | |
| --- | --- | --- | --- | --- | --- | --- | --- | --- | --- | --- | --- | --- | --- | --- | --- | --- | --- | --- | --- | --- | --- | --- | --- | --- | --- |
| Processing Level | | Minimally Processed Foods | | Culinary Ingredients | | Processed foods | | Ultra-processed foods | | Minimally Processed Foods | | Culinary Ingredients | | Processed foods | | Ultra-processed foods | | Minimally Processed Foods | | Culinary Ingredients | | Processed foods | | Ultra-processed foods | |
| Urbanicity | Year | **Mean** | SE | **Mean** | SE | **Mean** | SE | **Mean** | SE | **Mean** | SE | **Mean** | SE | **Mean** | SE | **Mean** | SE | **Mean** | SE | **Mean** | SE | **Mean** | SE | **Mean** | SE |
| Rural areas | 2006 | **76.1** | 1.6 | **0.9** | 0.2 | **11.6** | 1.2 | **11.4** | 0.9 | **83.4** | 1.8 | **5.0** | 0.8 | **5.4** | 0.6 | **6.2** | 1.3 | - | - | - | - | - | - | - | - |
|  | 2008 | **76.4** | 1.2 | **1.1** | 0.2 | **11.3** | 0.9 | **11.2** | 0.8 | **79.4** | 1.1 | **6.9** | 0.8 | **7.4** | 0.8 | **6.3** | 0.7 | - | - | - | - | - | - | - | - |
|  | 2010 | **75.4** | 1.5 | **1.2** | 0.2 | **11.6** | 1.0 | **11.9** | 1.1 | **80.3** | 1.2 | **6.8** | 1.0 | **6.4** | 0.6 | **6.4** | 1.0 | **66.1** | 1.9 | **4.2** | 0.8 | **22.3** | 1.8 | **7.4** | 0.8 |
|  | 2012 | **69.1** | 1.9 | **1.0** | 0.3 | **11.8** | 1.1 | **18.2** | 1.7 | **78.0** | 1.8 | **5.7** | 0.7 | **8.9** | 1.5 | **7.3** | 1.0 | **67.4** | 2.1 | **8.0** | 1.4 | **16.9** | 1.7 | **7.8** | 0.8 |
|  | 2014 | **71.7** | 1.5 | **1.4** | 0.3 | **12.1** | 1.0 | **14.8** | 1.0 | **81.9** | 1.7 | **4.5** | 0.9 | **7.4** | 1.0 | **6.2** | 1.0 | **63.3** | 2.0 | **6.3** | 1.2 | **21.4** | 1.6 | **9.0** | 1.1 |
|  | 2016 | **71.4** | 0.8 | **0.9** | 0.1 | **14.1** | 0.5 | **13.5** | 0.6 | **80.2** | 0.8 | **5.2** | 0.4 | **8.4** | 0.5 | **6.1** | 0.4 | **62.8** | 1.1 | **7.1** | 0.8 | **20.9** | 0.8 | **9.2** | 0.5 |
|  | 2018 | **72.0** | 0.7 | **0.8** | 0.1 | **14.8** | 0.5 | **12.5** | 0.4 | **80.7** | 0.7 | **4.8** | 0.3 | **9.1** | 0.5 | **5.5** | 0.3 | **62.9** | 0.9 | **7.1** | 0.6 | **22.1** | 0.8 | **7.9** | 0.4 |
|  | 2020 | **71.4** | 0.6 | **0.9** | 0.1 | **13.9** | 0.4 | **13.7** | 0.5 | **81.9** | 0.7 | **5.2** | 0.4 | **8.2** | 0.6 | **4.8** | 0.3 | **62.2** | 0.8 | **5.1** | 0.4 | **23.9** | 0.7 | **8.7** | 0.4 |
|  | 2022 | **72.3** | 0.6 | **0.9** | 0.1 | **14.9** | 0.4 | **11.9** | 0.4 | **80.5** | 0.7 | **4.7** | 0.3 | **9.0** | 0.4 | **5.9** | 0.4 | **62.4** | 0.8 | **5.2** | 0.4 | **24.1** | 0.7 | **8.3** | 0.4 |
| Small cities | 2006 | **70.5** | 3.7 | **1.2** | 0.6 | **14.2** | 1.9 | **14.2** | 2.5 | **86.2** | 3.2 | **3.0** | 1.0 | **6.9** | 1.4 | **3.9** | 1.4 | - | - | - | - | - | - | - | - |
|  | 2008 | **69.5** | 1.5 | **0.6** | 0.2 | **17.1** | 1.1 | **12.9** | 1.4 | **81.2** | 1.7 | **4.8** | 1.0 | **8.9** | 1.2 | **5.2** | 0.8 | - | - | - | - | - | - | - | - |
|  | 2010 | **68.8** | 2.7 | **0.7** | 0.2 | **17.3** | 1.8 | **13.2** | 1.6 | **82.8** | 1.5 | **4.3** | 0.8 | **7.8** | 0.9 | **5.0** | 1.0 | **60.3** | 3.5 | **1.5** | 0.4 | **27.5** | 3.7 | **10.6** | 1.3 |
|  | 2012 | **65.7** | 2.8 | **0.4** | 0.2 | **18.9** | 2.1 | **15.0** | 1.7 | **81.1** | 2.3 | **4.3** | 0.9 | **8.6** | 1.5 | **6.0** | 1.5 | **53.9** | 4.2 | **2.1** | 0.9 | **31.6** | 3.7 | **12.4** | 3.3 |
|  | 2014 | **67.4** | 2.1 | **0.5** | 0.2 | **17.7** | 1.4 | **14.3** | 1.4 | **82.2** | 1.8 | **2.9** | 0.8 | **10.6** | 1.6 | **4.3** | 0.8 | **61.9** | 2.5 | **2.1** | 1.1 | **25.9** | 2.2 | **10.2** | 1.5 |
|  | 2016 | **63.6** | 1.3 | **0.4** | 0.1 | **20.7** | 0.9 | **15.2** | 0.9 | **82.1** | 1.6 | **3.3** | 0.5 | **9.9** | 1.2 | **4.7** | 0.6 | **56.7** | 1.7 | **3.6** | 0.7 | **30.5** | 1.5 | **9.2** | 0.8 |
|  | 2018 | **63.1** | 1.2 | **0.4** | 0.1 | **21.9** | 1.0 | **14.7** | 0.7 | **80.2** | 1.2 | **2.9** | 0.3 | **12.3** | 1.1 | **4.6** | 0.6 | **54.9** | 2.1 | **2.0** | 0.4 | **33.0** | 1.8 | **10.2** | 1.1 |
|  | 2020 | **64.1** | 1.2 | **0.3** | 0.1 | **20.9** | 0.9 | **14.7** | 0.7 | **83.2** | 1.1 | **2.4** | 0.3 | **9.8** | 0.9 | **4.6** | 0.6 | **55.2** | 1.7 | **2.2** | 0.4 | **33.7** | 1.5 | **8.9** | 0.7 |
|  | 2022 | **64.6** | 1.1 | **0.5** | 0.1 | **21.7** | 0.9 | **13.1** | 0.7 | **81.5** | 0.9 | **4.1** | 0.5 | **10.2** | 1.0 | **4.2** | 0.5 | **54.9** | 1.3 | **2.6** | 0.5 | **32.8** | 1.3 | **9.7** | 0.6 |
| Medium-sized cities | 2006 | **69.6** | 1.5 | **0.4** | 0.1 | **18.9** | 1.2 | **11.1** | 0.9 | **82.4** | 1.6 | **2.6** | 0.5 | **10.6** | 1.4 | **4.3** | 0.6 | - | - | - | - | - | - | - | - |
|  | 2008 | **69.2** | 1.3 | **0.2** | 0.1 | **17.7** | 1.1 | **12.9** | 1.0 | **84.0** | 1.6 | **3.1** | 0.8 | **9.3** | 1.3 | **3.5** | 0.6 | - | - | - | - | - | - | - | - |
|  | 2010 | **63.6** | 1.7 | **0.3** | 0.1 | **21.8** | 1.4 | **14.3** | 1.1 | **82.1** | 2.2 | **2.5** | 0.5 | **11.8** | 2.3 | **3.6** | 0.8 | **55.7** | 3.1 | **1.4** | 0.7 | **33.5** | 2.8 | **9.4** | 1.3 |
|  | 2012 | **61.2** | 2.9 | **0.9** | 0.3 | **20.6** | 2.1 | **17.3** | 2.1 | **85.4** | 3.2 | **1.4** | 0.4 | **10.2** | 2.6 | **3.0** | 0.9 | **48.2** | 5.1 | **0.9** | 0.6 | **38.2** | 6.0 | **12.7** | 3.1 |
|  | 2014 | **61.0** | 2.3 | **0.7** | 0.3 | **22.0** | 1.6 | **16.3** | 1.4 | **83.8** | 1.8 | **2.5** | 0.7 | **8.0** | 1.0 | **5.7** | 1.3 | **60.1** | 3.7 | **0.8** | 0.3 | **25.9** | 3.0 | **13.1** | 2.0 |
|  | 2016 | **59.8** | 1.4 | **0.3** | 0.1 | **25.5** | 1.1 | **14.4** | 0.8 | **82.3** | 1.6 | **1.8** | 0.4 | **12.2** | 1.4 | **3.7** | 0.5 | **46.1** | 2.1 | **2.2** | 0.7 | **39.2** | 2.0 | **12.5** | 1.3 |
|  | 2018 | **61.4** | 1.5 | **0.5** | 0.1 | **24.4** | 1.0 | **13.7** | 0.9 | **81.2** | 1.4 | **2.0** | 0.5 | **11.2** | 1.1 | **5.6** | 0.8 | **45.4** | 2.1 | **2.6** | 0.7 | **38.8** | 2.0 | **13.2** | 1.6 |
|  | 2020 | **61.5** | 1.2 | **0.4** | 0.1 | **24.0** | 0.9 | **14.1** | 0.7 | **84.2** | 1.0 | **2.2** | 0.3 | **10.0** | 0.8 | **3.6** | 0.4 | **46.6** | 1.8 | **1.7** | 0.4 | **40.5** | 1.5 | **11.2** | 1.0 |
|  | 2022 | **56.9** | 1.3 | **0.4** | 0.1 | **27.5** | 1.0 | **15.3** | 0.9 | **82.4** | 1.2 | **2.1** | 0.3 | **10.8** | 1.2 | **4.7** | 0.6 | **48.1** | 1.7 | **1.3** | 0.3 | **42.6** | 1.6 | **8.1** | 0.7 |
| Metropolitan cities | 2006 | **64.5** | 1.0 | **0.3** | 0.1 | **23.7** | 0.8 | **11.5** | 0.6 | **83.0** | 0.8 | **2.1** | 0.3 | **9.8** | 0.6 | **5.0** | 0.5 | - | - | - | - | - | - | - | - |
|  | 2008 | **63.8** | 0.8 | **0.3** | 0.1 | **22.3** | 0.6 | **13.7** | 0.5 | **81.9** | 0.7 | **2.3** | 0.2 | **11.8** | 0.7 | **4.0** | 0.3 | - | - | - | - | - | - | - | - |
|  | 2010 | **60.5** | 1.0 | **0.4** | 0.1 | **24.5** | 0.8 | **14.6** | 0.7 | **81.7** | 0.8 | **2.5** | 0.2 | **10.7** | 0.7 | **5.1** | 0.4 | **41.1** | 2.0 | **0.7** | 0.3 | **43.6** | 2.0 | **14.6** | 1.4 |
|  | 2012 | **63.0** | 2.0 | **0.1** | 0.1 | **22.6** | 1.5 | **14.3** | 1.5 | **82.6** | 1.4 | **3.0** | 0.5 | **8.8** | 1.0 | **5.5** | 0.9 | **32.1** | 2.9 | **1.3** | 0.7 | **50.2** | 3.2 | **16.4** | 2.4 |
|  | 2014 | **58.5** | 1.4 | **0.6** | 0.2 | **26.6** | 1.1 | **14.3** | 0.9 | **81.6** | 0.9 | **1.6** | 0.2 | **12.3** | 0.8 | **4.5** | 0.5 | **36.2** | 2.2 | **1.1** | 0.3 | **48.7** | 2.1 | **14.0** | 1.5 |
|  | 2016 | **55.8** | 0.8 | **0.4** | 0.1 | **30.4** | 0.7 | **13.4** | 0.5 | **80.0** | 0.7 | **1.9** | 0.2 | **14.3** | 0.6 | **3.8** | 0.2 | **36.9** | 1.2 | **1.1** | 0.3 | **49.3** | 1.2 | **12.7** | 0.8 |
|  | 2018 | **56.3** | 0.9 | **0.4** | 0.1 | **29.4** | 0.7 | **13.9** | 0.5 | **80.9** | 0.7 | **1.5** | 0.1 | **13.2** | 0.6 | **4.5** | 0.3 | **36.6** | 1.3 | **1.3** | 0.3 | **49.7** | 1.3 | **12.4** | 0.9 |
|  | 2020 | **58.9** | 0.7 | **0.5** | 0.1 | **28.2** | 0.6 | **12.4** | 0.4 | **83.1** | 0.6 | **1.6** | 0.1 | **11.4** | 0.5 | **4.0** | 0.3 | **38.2** | 1.0 | **1.1** | 0.2 | **47.8** | 1.0 | **13.0** | 0.6 |
|  | 2022 | **54.3** | 0.7 | **0.3** | 0.1 | **32.6** | 0.7 | **12.7** | 0.5 | **80.8** | 0.6 | **1.7** | 0.1 | **12.9** | 0.5 | **4.7** | 0.3 | **35.5** | 1.1 | **0.9** | 0.2 | **51.2** | 1.1 | **12.4** | 0.7 |

**Table 5.2. Trends in the proportion of food and beverage purchases by processing level in traditional outlets stratified by urbanicity, Data from ENIGH 2006 to 2022.**

| Food outlet | | Public Markets | | | | | | | | Specialty stores | | | | | | | | Small neighborhood stores | | | | | | | |
| --- | --- | --- | --- | --- | --- | --- | --- | --- | --- | --- | --- | --- | --- | --- | --- | --- | --- | --- | --- | --- | --- | --- | --- | --- | --- |
| Processing Level | | Minimally Processed Foods | | Culinary Ingredients | | Processed foods | | Ultra-processed foods | | Minimally Processed Foods | | Culinary Ingredients | | Processed foods | | Ultra-processed foods | | Minimally Processed Foods | | Culinary Ingredients | | Processed foods | | Ultra-processed foods | |
| Urbanicity | Year | **Mean** | SE | **Mean** | SE | **Mean** | SE | **Mean** | SE | **Mean** | SE | **Mean** | SE | **Mean** | SE | **Mean** | SE | **Mean** | SE | **Mean** | SE | **Mean** | SE | **Mean** | SE |
| Rural areas | 2006 | **82.2** | 1.8 | **4.6** | 0.7 | **7.3** | 1.0 | **5.9** | 1.0 | **72.9** | 1.9 | **10.0** | 1.4 | **8.4** | 1.0 | **8.7** | 1.0 | **48.8** | 0.8 | **11.5** | 0.6 | **8.4** | 0.4 | **31.3** | 0.8 |
|  | 2008 | **81.8** | 1.1 | **3.5** | 0.4 | **8.1** | 0.6 | **6.7** | 0.7 | **72.4** | 1.8 | **14.1** | 1.5 | **5.3** | 0.4 | **8.2** | 0.6 | **50.6** | 0.7 | **13.3** | 0.5 | **8.7** | 0.4 | **27.5** | 0.6 |
|  | 2010 | **80.7** | 1.1 | **4.5** | 0.5 | **7.4** | 0.6 | **7.4** | 0.6 | **71.3** | 2.1 | **13.2** | 2.1 | **7.3** | 0.8 | **8.3** | 0.7 | **52.1** | 0.8 | **9.8** | 0.4 | **9.6** | 0.5 | **28.4** | 0.7 |
|  | 2012 | **81.2** | 1.3 | **3.4** | 0.6 | **7.1** | 0.8 | **8.3** | 0.9 | **69.4** | 2.2 | **14.2** | 2.5 | **8.0** | 0.7 | **8.4** | 0.8 | **50.7** | 1.0 | **9.9** | 0.5 | **9.0** | 0.4 | **30.3** | 0.9 |
|  | 2014 | **78.3** | 1.3 | **4.1** | 0.5 | **9.0** | 0.9 | **8.6** | 0.9 | **70.3** | 1.7 | **13.0** | 1.6 | **7.8** | 0.6 | **8.8** | 0.7 | **49.1** | 0.8 | **9.2** | 0.4 | **9.7** | 0.4 | **31.9** | 0.8 |
|  | 2016 | **80.6** | 0.6 | **3.5** | 0.3 | **9.0** | 0.4 | **6.9** | 0.4 | **74.4** | 0.8 | **9.9** | 0.7 | **7.3** | 0.2 | **8.4** | 0.3 | **50.3** | 0.4 | **9.4** | 0.2 | **10.1** | 0.2 | **30.3** | 0.4 |
|  | 2018 | **79.3** | 0.6 | **3.9** | 0.2 | **8.9** | 0.4 | **7.9** | 0.4 | **75.9** | 0.6 | **7.9** | 0.6 | **8.4** | 0.2 | **7.7** | 0.2 | **50.3** | 0.4 | **9.4** | 0.2 | **10.2** | 0.2 | **30.2** | 0.4 |
|  | 2020 | **79.2** | 0.6 | **4.0** | 0.3 | **9.3** | 0.4 | **7.5** | 0.4 | **75.9** | 0.5 | **7.1** | 0.5 | **9.3** | 0.2 | **7.7** | 0.2 | **50.7** | 0.3 | **9.2** | 0.2 | **9.7** | 0.2 | **30.3** | 0.3 |
|  | 2022 | **79.2** | 0.5 | **3.9** | 0.2 | **9.1** | 0.4 | **7.9** | 0.4 | **76.2** | 0.4 | **5.6** | 0.4 | **10.1** | 0.2 | **8.1** | 0.2 | **49.4** | 0.3 | **9.0** | 0.1 | **10.7** | 0.2 | **30.9** | 0.3 |
| Small cities | 2006 | **81.7** | 1.7 | **3.1** | 0.6 | **7.1** | 0.8 | **8.1** | 1.5 | **79.4** | 1.6 | **5.9** | 1.3 | **7.9** | 0.6 | **6.8** | 0.8 | **49.2** | 1.5 | **9.0** | 0.6 | **9.2** | 0.8 | **32.5** | 1.2 |
|  | 2008 | **79.3** | 1.5 | **3.3** | 0.4 | **11.3** | 1.2 | **6.1** | 0.7 | **79.4** | 1.2 | **4.4** | 1.1 | **7.1** | 0.5 | **9.1** | 0.9 | **51.7** | 0.7 | **9.0** | 0.4 | **10.0** | 0.4 | **29.3** | 0.7 |
|  | 2010 | **82.9** | 1.2 | **2.5** | 0.3 | **9.5** | 1.1 | **5.1** | 0.6 | **79.7** | 1.4 | **4.2** | 1.3 | **7.4** | 0.5 | **8.7** | 0.6 | **50.2** | 0.9 | **8.6** | 0.4 | **10.3** | 0.4 | **30.9** | 0.9 |
|  | 2012 | **78.7** | 1.9 | **2.8** | 0.6 | **10.3** | 1.4 | **8.2** | 1.0 | **77.3** | 1.7 | **3.9** | 1.3 | **9.6** | 1.0 | **9.2** | 1.0 | **47.9** | 1.3 | **7.3** | 0.6 | **10.9** | 0.7 | **33.8** | 1.3 |
|  | 2014 | **78.7** | 1.8 | **3.0** | 0.5 | **11.5** | 1.5 | **6.8** | 0.9 | **77.9** | 1.2 | **4.1** | 0.9 | **9.4** | 0.7 | **8.6** | 0.7 | **50.2** | 0.8 | **7.1** | 0.4 | **12.4** | 0.6 | **30.4** | 0.8 |
|  | 2016 | **78.7** | 1.1 | **2.8** | 0.3 | **12.2** | 0.9 | **6.3** | 0.5 | **79.6** | 0.7 | **2.3** | 0.4 | **9.4** | 0.4 | **8.7** | 0.5 | **50.1** | 0.6 | **7.6** | 0.2 | **11.7** | 0.3 | **30.6** | 0.5 |
|  | 2018 | **79.6** | 0.9 | **2.6** | 0.3 | **11.6** | 0.7 | **6.2** | 0.5 | **78.9** | 0.6 | **2.1** | 0.3 | **10.6** | 0.4 | **8.4** | 0.4 | **50.4** | 0.5 | **7.3** | 0.3 | **12.2** | 0.4 | **30.1** | 0.6 |
|  | 2020 | **79.2** | 1.0 | **2.4** | 0.2 | **11.6** | 0.7 | **6.8** | 0.7 | **77.6** | 0.5 | **2.3** | 0.3 | **11.3** | 0.4 | **8.8** | 0.3 | **51.0** | 0.5 | **7.6** | 0.2 | **11.6** | 0.3 | **29.8** | 0.5 |
|  | 2022 | **78.0** | 0.9 | **2.1** | 0.2 | **12.9** | 0.7 | **7.1** | 0.5 | **77.5** | 0.5 | **1.7** | 0.2 | **11.6** | 0.3 | **9.2** | 0.3 | **49.7** | 0.5 | **7.4** | 0.2 | **12.1** | 0.2 | **30.8** | 0.4 |
| Medium-sized cities | 2006 | **80.0** | 1.0 | **2.4** | 0.3 | **11.0** | 0.7 | **6.6** | 0.6 | **77.8** | 1.0 | **0.8** | 0.1 | **10.8** | 0.7 | **10.6** | 0.6 | **45.8** | 0.8 | **6.5** | 0.6 | **9.8** | 0.4 | **37.8** | 0.6 |
|  | 2008 | **79.1** | 1.1 | **2.8** | 0.3 | **12.2** | 0.9 | **6.0** | 0.6 | **79.9** | 0.8 | **0.8** | 0.1 | **9.3** | 0.5 | **10.0** | 0.6 | **47.8** | 0.6 | **6.1** | 0.4 | **11.2** | 0.4 | **34.9** | 0.7 |
|  | 2010 | **82.5** | 1.0 | **2.2** | 0.2 | **9.5** | 0.6 | **5.8** | 0.6 | **78.3** | 0.9 | **1.0** | 0.1 | **10.0** | 0.6 | **10.6** | 0.5 | **48.1** | 0.9 | **5.8** | 0.3 | **10.8** | 0.5 | **35.3** | 0.9 |
|  | 2012 | **76.9** | 1.8 | **2.2** | 0.4 | **11.9** | 1.3 | **8.9** | 1.2 | **76.1** | 1.6 | **1.0** | 0.3 | **12.0** | 0.9 | **10.9** | 1.1 | **48.1** | 1.4 | **5.9** | 0.6 | **10.8** | 0.6 | **35.2** | 1.4 |
|  | 2014 | **81.7** | 1.2 | **2.1** | 0.3 | **10.7** | 0.9 | **5.4** | 0.7 | **75.9** | 1.2 | **1.0** | 0.2 | **11.2** | 0.7 | **11.9** | 0.9 | **50.5** | 1.0 | **5.0** | 0.3 | **11.5** | 0.6 | **32.9** | 0.9 |
|  | 2016 | **80.2** | 0.7 | **1.9** | 0.2 | **11.8** | 0.6 | **6.1** | 0.4 | **75.9** | 0.6 | **0.9** | 0.1 | **12.0** | 0.4 | **11.1** | 0.4 | **47.7** | 0.6 | **6.1** | 0.2 | **11.2** | 0.3 | **35.0** | 0.6 |
|  | 2018 | **78.8** | 0.7 | **2.5** | 0.2 | **12.7** | 0.6 | **6.0** | 0.4 | **76.2** | 0.6 | **0.7** | 0.1 | **12.5** | 0.4 | **10.5** | 0.4 | **47.8** | 0.5 | **5.9** | 0.2 | **12.0** | 0.4 | **34.3** | 0.5 |
|  | 2020 | **79.2** | 0.7 | **2.4** | 0.2 | **13.0** | 0.6 | **5.3** | 0.3 | **74.5** | 0.5 | **0.9** | 0.1 | **13.8** | 0.4 | **10.8** | 0.3 | **48.9** | 0.5 | **6.2** | 0.2 | **11.1** | 0.3 | **33.8** | 0.5 |
|  | 2022 | **78.9** | 0.6 | **2.2** | 0.2 | **12.7** | 0.5 | **6.2** | 0.4 | **73.8** | 0.5 | **0.8** | 0.1 | **14.4** | 0.4 | **11.0** | 0.3 | **48.3** | 0.5 | **6.3** | 0.2 | **11.6** | 0.2 | **33.8** | 0.5 |
| Metropolitan cities | 2006 | **79.9** | 0.6 | **2.0** | 0.1 | **11.2** | 0.4 | **7.0** | 0.3 | **72.8** | 0.5 | **0.4** | 0.0 | **12.8** | 0.4 | **14.0** | 0.4 | **43.5** | 0.4 | **4.1** | 0.1 | **11.0** | 0.2 | **41.4** | 0.4 |
|  | 2008 | **78.1** | 0.5 | **2.7** | 0.1 | **11.5** | 0.4 | **7.7** | 0.3 | **73.4** | 0.4 | **0.5** | 0.0 | **12.8** | 0.3 | **13.2** | 0.3 | **46.1** | 0.4 | **4.2** | 0.1 | **11.2** | 0.2 | **38.5** | 0.4 |
|  | 2010 | **78.4** | 0.5 | **2.7** | 0.2 | **11.0** | 0.4 | **7.9** | 0.3 | **73.3** | 0.5 | **0.6** | 0.1 | **12.2** | 0.3 | **13.9** | 0.3 | **46.2** | 0.4 | **4.4** | 0.1 | **11.2** | 0.2 | **38.2** | 0.4 |
|  | 2012 | **78.8** | 1.2 | **2.2** | 0.4 | **11.1** | 1.0 | **7.9** | 0.7 | **70.2** | 1.0 | **0.4** | 0.1 | **14.3** | 0.7 | **15.1** | 0.8 | **43.9** | 0.8 | **4.0** | 0.3 | **11.6** | 0.4 | **40.4** | 0.8 |
|  | 2014 | **78.4** | 0.7 | **2.1** | 0.2 | **12.7** | 0.6 | **6.7** | 0.4 | **70.9** | 0.6 | **0.6** | 0.1 | **13.8** | 0.4 | **14.8** | 0.5 | **45.6** | 0.5 | **4.4** | 0.2 | **12.3** | 0.3 | **37.7** | 0.5 |
|  | 2016 | **78.5** | 0.5 | **2.3** | 0.1 | **11.5** | 0.3 | **7.8** | 0.3 | **69.8** | 0.3 | **0.6** | 0.0 | **14.5** | 0.2 | **15.1** | 0.3 | **44.0** | 0.3 | **4.5** | 0.1 | **12.5** | 0.2 | **38.9** | 0.3 |
|  | 2018 | **78.5** | 0.5 | **2.3** | 0.1 | **11.8** | 0.4 | **7.3** | 0.3 | **68.7** | 0.4 | **0.5** | 0.0 | **15.7** | 0.3 | **15.0** | 0.3 | **44.9** | 0.3 | **4.8** | 0.1 | **12.9** | 0.2 | **37.4** | 0.3 |
|  | 2020 | **78.8** | 0.4 | **2.5** | 0.1 | **11.0** | 0.3 | **7.7** | 0.3 | **69.2** | 0.3 | **0.6** | 0.0 | **16.3** | 0.2 | **14.0** | 0.2 | **46.2** | 0.3 | **5.1** | 0.1 | **11.9** | 0.2 | **36.8** | 0.3 |
|  | 2022 | **78.1** | 0.4 | **2.6** | 0.1 | **11.6** | 0.3 | **7.7** | 0.2 | **68.3** | 0.3 | **0.6** | 0.0 | **16.6** | 0.2 | **14.6** | 0.2 | **45.0** | 0.3 | **5.0** | 0.1 | **12.7** | 0.2 | **37.2** | 0.3 |

**Table 5.3. Trends in the proportion of food and beverage purchases by processing level in supermarkets, chain convenience stores and other outlets, stratified by urbanicity, Data from ENIGH 2006 to 2022.**

| Food outlet | | Supermarkets | | | | | | | | Chain convenience stores | | | | | | | | Other stores | | | | | | | |
| --- | --- | --- | --- | --- | --- | --- | --- | --- | --- | --- | --- | --- | --- | --- | --- | --- | --- | --- | --- | --- | --- | --- | --- | --- | --- |
| Processing Level | | Minimally Processed Foods | | Culinary Ingredients | | Processed foods | | Ultra-processed foods | | Minimally Processed Foods | | Culinary Ingredients | | Processed foods | | Ultra-processed foods | | Minimally Processed Foods | | Culinary Ingredients | | Processed foods | | Ultra-processed foods | |
| Urbanicity | Year | **Mean** | SE | **Mean** | SE | **Mean** | SE | **Mean** | SE | **Mean** | SE | **Mean** | SE | **Mean** | SE | **Mean** | SE | **Mean** | SE | **Mean** | SE | **Mean** | SE | **Mean** | SE |
| Rural areas | 2006 | **43.7** | 3.5 | **11.3** | 2.1 | **6.9** | 0.9 | **38.0** | 4.2 | **27.2** | 7.5 | **1.3** | 0.7 | **9.2** | 4.8 | **62.3** | 9.3 | **64.3** | 1.9 | **3.7** | 0.8 | **21.1** | 2.6 | **10.9** | 2.3 |
|  | 2008 | **48.8** | 1.6 | **13.3** | 1.2 | **7.6** | 0.8 | **30.2** | 1.6 | **23.0** | 6.4 | **10.8** | 7.9 | **23.8** | 8.2 | **42.3** | 7.6 | **64.3** | 3.1 | **4.8** | 1.5 | **19.5** | 2.5 | **11.4** | 1.6 |
|  | 2010 | **48.1** | 2.3 | **10.3** | 1.1 | **9.0** | 0.9 | **32.6** | 1.9 | **49.7** | 8.0 | **1.5** | 0.6 | **19.7** | 5.7 | **29.2** | 5.4 | **54.0** | 1.6 | **24.1** | 1.5 | **5.9** | 0.7 | **16.1** | 1.2 |
|  | 2012 | **50.1** | 2.6 | **8.4** | 1.0 | **8.1** | 1.2 | **33.4** | 2.0 | **32.0** | 5.7 | **0.9** | 0.9 | **7.2** | 3.8 | **59.9** | 5.9 | **50.8** | 2.2 | **25.0** | 1.7 | **4.7** | 0.8 | **19.6** | 1.3 |
|  | 2014 | **47.7** | 1.8 | **11.6** | 0.9 | **9.3** | 0.7 | **31.4** | 1.7 | **32.8** | 4.1 | **1.7** | 1.0 | **11.0** | 2.9 | **54.6** | 4.6 | **53.6** | 1.8 | **17.7** | 1.4 | **6.1** | 0.8 | **22.6** | 1.5 |
|  | 2016 | **47.1** | 0.8 | **10.8** | 0.5 | **10.0** | 0.5 | **32.0** | 0.8 | **34.5** | 2.9 | **3.5** | 0.8 | **12.9** | 2.0 | **49.1** | 3.7 | **53.2** | 1.1 | **20.3** | 0.8 | **5.9** | 0.3 | **20.6** | 0.8 |
|  | 2018 | **48.8** | 0.8 | **11.6** | 0.5 | **9.4** | 0.4 | **30.2** | 0.7 | **31.8** | 1.8 | **5.5** | 1.0 | **14.5** | 1.4 | **48.1** | 2.2 | **55.0** | 1.0 | **19.7** | 0.8 | **6.7** | 0.4 | **18.6** | 0.7 |
|  | 2020 | **52.2** | 0.8 | **11.5** | 0.5 | **9.2** | 0.4 | **27.1** | 0.7 | **33.9** | 1.6 | **6.9** | 0.9 | **15.8** | 1.3 | **43.5** | 1.8 | **56.9** | 0.9 | **19.9** | 0.8 | **5.6** | 0.4 | **17.5** | 0.7 |
|  | 2022 | **48.3** | 0.7 | **12.8** | 0.5 | **9.3** | 0.4 | **29.6** | 0.6 | **32.2** | 1.4 | **7.9** | 1.0 | **14.5** | 1.1 | **45.4** | 1.6 | **52.8** | 0.9 | **22.3** | 0.8 | **5.8** | 0.3 | **19.1** | 0.6 |
| Small cities | 2006 | **31.9** | 3.6 | **9.9** | 2.6 | **14.9** | 3.3 | **43.3** | 2.5 | **45.8** | 18.0 | **0.0** | 0.0 | **31.7** | 16.2 | **22.5** | 12.8 | **64.9** | 6.1 | **3.4** | 1.1 | **20.1** | 4.0 | **11.6** | 2.6 |
|  | 2008 | **45.4** | 3.4 | **7.9** | 1.1 | **12.1** | 3.1 | **34.7** | 3.0 | **45.7** | 16.3 | **0.1** | 0.1 | **15.6** | 9.2 | **38.6** | 13.5 | **47.9** | 4.3 | **6.1** | 3.6 | **26.7** | 5.5 | **19.3** | 4.1 |
|  | 2010 | **46.9** | 2.3 | **12.4** | 2.1 | **9.1** | 1.9 | **31.6** | 2.5 | **34.2** | 11.0 | **0.7** | 0.5 | **14.4** | 5.9 | **50.6** | 10.4 | **59.1** | 3.0 | **12.8** | 1.8 | **5.4** | 1.0 | **22.7** | 3.3 |
|  | 2012 | **43.8** | 3.3 | **6.9** | 1.6 | **9.4** | 1.4 | **39.9** | 3.7 | **33.0** | 4.7 | **2.8** | 1.2 | **16.9** | 5.0 | **47.3** | 4.6 | **48.2** | 5.6 | **6.2** | 1.7 | **4.0** | 1.7 | **41.6** | 5.9 |
|  | 2014 | **50.8** | 2.0 | **7.6** | 1.1 | **10.3** | 0.9 | **31.2** | 1.8 | **37.3** | 5.5 | **6.1** | 4.1 | **9.7** | 2.4 | **46.9** | 6.2 | **66.6** | 3.8 | **8.9** | 2.8 | **4.1** | 1.1 | **20.4** | 2.8 |
|  | 2016 | **50.9** | 1.5 | **7.2** | 0.6 | **10.4** | 0.6 | **31.5** | 1.3 | **30.4** | 2.9 | **5.3** | 1.1 | **18.3** | 2.2 | **46.0** | 2.9 | **64.8** | 2.5 | **9.1** | 1.0 | **7.0** | 1.1 | **19.1** | 1.8 |
|  | 2018 | **51.0** | 1.2 | **7.2** | 0.6 | **10.4** | 0.6 | **31.3** | 1.0 | **29.8** | 2.5 | **5.2** | 1.1 | **18.6** | 2.1 | **46.4** | 2.9 | **62.7** | 2.6 | **9.0** | 1.4 | **8.7** | 1.6 | **19.6** | 2.0 |
|  | 2020 | **53.3** | 1.2 | **9.4** | 0.8 | **10.2** | 0.6 | **27.1** | 1.0 | **33.8** | 2.3 | **4.0** | 0.7 | **16.5** | 1.7 | **45.7** | 2.3 | **69.2** | 2.3 | **11.4** | 2.0 | **6.5** | 1.2 | **12.9** | 1.3 |
|  | 2022 | **48.7** | 1.3 | **11.8** | 1.0 | **11.2** | 0.7 | **28.3** | 1.0 | **31.4** | 2.1 | **6.8** | 1.1 | **14.2** | 1.5 | **47.6** | 2.1 | **62.7** | 2.4 | **7.8** | 1.0 | **10.5** | 1.6 | **19.1** | 1.7 |
| Medium-sized cities | 2006 | **47.6** | 1.4 | **6.3** | 0.5 | **9.6** | 0.7 | **36.5** | 1.2 | **38.1** | 5.4 | **7.0** | 4.9 | **7.8** | 2.6 | **47.1** | 4.9 | **45.9** | 4.3 | **1.3** | 0.5 | **32.2** | 4.4 | **20.6** | 3.0 |
|  | 2008 | **49.1** | 1.3 | **7.7** | 0.7 | **9.1** | 0.5 | **34.0** | 1.3 | **21.6** | 5.5 | **0.6** | 0.3 | **27.7** | 13.2 | **50.1** | 9.1 | **41.7** | 3.0 | **1.3** | 0.6 | **43.8** | 3.8 | **13.2** | 2.4 |
|  | 2010 | **49.8** | 1.3 | **6.5** | 0.5 | **11.0** | 0.9 | **32.7** | 1.2 | **29.1** | 4.0 | **4.4** | 1.3 | **11.2** | 2.5 | **55.2** | 4.1 | **49.7** | 5.8 | **6.6** | 2.6 | **10.0** | 3.2 | **33.7** | 6.5 |
|  | 2012 | **45.2** | 2.5 | **7.3** | 1.1 | **11.5** | 1.6 | **36.1** | 2.2 | **20.4** | 4.3 | **4.2** | 3.5 | **17.9** | 4.9 | **57.5** | 5.8 | **53.4** | 12.6 | **5.6** | 2.4 | **10.1** | 5.2 | **30.9** | 10.7 |
|  | 2014 | **51.6** | 1.2 | **6.9** | 0.5 | **11.6** | 1.1 | **29.9** | 1.2 | **33.1** | 3.4 | **2.8** | 1.0 | **16.2** | 3.7 | **47.9** | 4.3 | **74.0** | 4.9 | **5.2** | 1.9 | **3.2** | 1.1 | **17.6** | 3.8 |
|  | 2016 | **51.0** | 0.9 | **7.3** | 0.4 | **10.6** | 0.4 | **31.0** | 0.8 | **34.6** | 1.9 | **2.5** | 0.4 | **12.7** | 1.3 | **50.2** | 1.9 | **56.1** | 3.6 | **6.0** | 1.3 | **10.9** | 2.1 | **27.0** | 2.7 |
|  | 2018 | **53.3** | 0.9 | **7.5** | 0.4 | **10.7** | 0.5 | **28.6** | 0.8 | **34.0** | 2.0 | **3.4** | 0.7 | **16.1** | 1.5 | **46.5** | 1.8 | **57.5** | 3.4 | **3.8** | 0.9 | **14.5** | 2.2 | **24.3** | 2.5 |
|  | 2020 | **55.0** | 1.0 | **7.1** | 0.4 | **10.8** | 0.5 | **27.1** | 0.8 | **33.4** | 1.6 | **3.6** | 0.5 | **14.4** | 1.2 | **48.7** | 1.6 | **62.4** | 3.1 | **5.7** | 1.2 | **10.6** | 1.9 | **21.3** | 2.7 |
|  | 2022 | **53.9** | 0.9 | **8.3** | 0.6 | **10.7** | 0.4 | **27.1** | 0.7 | **30.9** | 1.4 | **4.5** | 0.6 | **14.5** | 1.6 | **50.0** | 1.8 | **54.8** | 2.8 | **5.9** | 1.5 | **13.6** | 2.1 | **25.8** | 2.6 |
| Metropolitan cities | 2006 | **50.4** | 0.6 | **5.1** | 0.2 | **10.3** | 0.3 | **34.2** | 0.6 | **35.6** | 2.2 | **1.6** | 0.5 | **13.8** | 1.4 | **49.1** | 2.2 | **37.2** | 2.0 | **2.0** | 0.4 | **35.8** | 2.0 | **25.0** | 1.7 |
|  | 2008 | **51.6** | 0.5 | **5.9** | 0.2 | **11.1** | 0.3 | **31.5** | 0.5 | **33.4** | 1.7 | **2.0** | 0.5 | **17.1** | 1.5 | **47.6** | 1.9 | **39.9** | 2.3 | **2.0** | 0.4 | **34.5** | 1.9 | **23.5** | 1.8 |
|  | 2010 | **51.7** | 0.6 | **5.6** | 0.2 | **11.2** | 0.3 | **31.6** | 0.5 | **38.3** | 1.7 | **2.4** | 0.5 | **11.9** | 1.1 | **47.4** | 1.7 | **54.2** | 2.9 | **3.6** | 0.9 | **10.6** | 1.8 | **31.6** | 2.6 |
|  | 2012 | **52.3** | 1.0 | **4.8** | 0.3 | **11.0** | 0.6 | **32.0** | 0.9 | **34.5** | 2.3 | **2.3** | 0.6 | **13.5** | 1.5 | **49.7** | 2.3 | **57.2** | 6.1 | **3.9** | 1.4 | **9.1** | 2.1 | **29.9** | 4.8 |
|  | 2014 | **53.4** | 0.6 | **5.2** | 0.2 | **11.7** | 0.4 | **29.7** | 0.6 | **32.6** | 1.3 | **2.7** | 0.5 | **13.8** | 0.9 | **51.0** | 1.3 | **58.4** | 2.8 | **4.8** | 0.9 | **11.4** | 1.8 | **25.5** | 2.3 |
|  | 2016 | **52.3** | 0.4 | **5.5** | 0.1 | **11.7** | 0.3 | **30.6** | 0.4 | **32.4** | 0.7 | **3.0** | 0.3 | **12.5** | 0.5 | **52.2** | 0.8 | **52.0** | 2.0 | **4.2** | 0.7 | **14.0** | 1.3 | **29.8** | 1.7 |
|  | 2018 | **54.8** | 0.4 | **5.1** | 0.1 | **11.5** | 0.3 | **28.6** | 0.4 | **32.5** | 0.7 | **2.6** | 0.2 | **14.1** | 0.5 | **50.9** | 0.7 | **56.1** | 2.0 | **3.4** | 0.8 | **14.0** | 1.4 | **26.5** | 1.6 |
|  | 2020 | **57.0** | 0.4 | **5.8** | 0.1 | **10.7** | 0.2 | **26.5** | 0.3 | **31.8** | 0.7 | **3.7** | 0.2 | **14.2** | 0.5 | **50.3** | 0.7 | **59.5** | 1.6 | **4.7** | 0.7 | **14.2** | 1.1 | **21.6** | 1.2 |
|  | 2022 | **55.3** | 0.4 | **6.7** | 0.2 | **11.3** | 0.2 | **26.7** | 0.3 | **29.0** | 0.6 | **3.8** | 0.3 | **13.9** | 0.5 | **53.3** | 0.7 | **51.6** | 1.5 | **3.5** | 0.5 | **17.8** | 1.1 | **27.1** | 1.4 |
